# Climate Spaces and Cliffs: A Novel Bovine Thermodynamic and Mass Balances Model

**DOI:** 10.3390/ani13193043

**Published:** 2023-09-27

**Authors:** Warren P. Porter, Alexa E. Bertz, Paul D. Mathewson, Luis C. Solorzano, Peter N. Dudley, Riccardo Bonazza, Kifle G. Gebremedhin

**Affiliations:** 1 Department of Integrative Biology, University of Wisconsin, Madison, WI 53706, USA; abertz@wisc.edu (A.E.B.); mathewson@wisc.edu (P.D.M.); 2 Adjunct Faculty, Department of Animal Science, University of Puerto Rico-Mayagüez, Mayagüez, PR 00680, USA; lankinusa@gmail.com; 3 Fisheries Collaborative Program, University of California, Santa Cruz, CA 95064, USA; pndudley@ucsc.edu; 4 Department of Mechanical Engineering, University of Wisconsin, Madison, WI 53706, USA; riccardo.bonazza@wisc.edu; 5 Department of Biological and Environmental Engineering, Cornell University, Ithaca, NY 14853, USA; kgg1@cornell.edu

**Keywords:** climate change, mechanistic microclimate-cow models, milk production, climate cliffs

## Abstract

**Simple Summary:**

Climate change is causing an increase in air temperature, and consequently, animals are increasingly subject to heat stress, which is responsible for causing changes in their physiological and behavioral reactions, as well as reductions in feed intake, efficiency, growth, reproduction and milk and meat production. There are not only climate spaces for daily functions, but also climate cliffs that cause reproductive failures in the face of climate warming. The objective of this study was to develop a state-of-the-art model that calculates the impacts of climate and animal variables on milk production, metabolic rate, feed consumption and water needs. The study identifies current and future monthly latitudinal climate change impacts on milk production and feed and water needs in dairy cows on high-grain versus high-forage diets at three arbitrary north latitudes, 12°, 30° and 60°, for North and Central America and Asia. These three latitudes encompass current northern hemisphere bovine production environments and possible future production locations. The greatest impacts of climate change will be in the low elevations in tropical and subtropical regions. Global regions above 30° and below 60° latitude with reliable rainfall will be least affected by current projected levels of climate change.

**Abstract:**

The effects of climate change on animals are typically viewed in terms of survivability and wellbeing. In this study, we broaden that purview to include climate impacts on reproductive capability. There are not only climate spaces for daily function, but climate cliffs that represent reproductive failures in the face of climate warming. This alternative focus suggests that climate warming challenges may be more immediate and profound than initially imagined. This research describes a state-of-the-art mechanistic model, Dairy Niche Mapper (DNM), and independent validation tests. Where test data are absent, the calculated results are consistent with expected responses. Simulations of metabolic chamber conditions reveal the local steady-state impacts of climate and animal variables on milk production capacity, metabolic rate, food consumption and water needs. Simulations of a temperature humidity index (THI) show strengths and limitations of that approach. Broader time- and spatial-scale calculations applied in the western and eastern halves of the northern hemisphere identify current and future monthly latitudinal climate change impacts on milk production potential, feed and water needs in dairy cows of different sizes. Dairy Niche Mapper (DNM) was developed from a broadly tested mechanistic microclimate-animal model, Niche Mapper (NM). DNM provides an improved quantitative understanding of the complex nonlinear interactions of climate variation and dairy bovine properties’ effects on current and future milk production, feed and water needs for grazing and confinement dairy operations. DNM outputs include feasible activity times, milk production and water and feed needs of different-sized Holstein cows on high-grain (confinement feeding) versus high-forage (grazing feeding) diets at three arbitrary north latitudes, 12°, 30° and 60°, for North and Central America and for Asia. These three latitudes encompass current northern hemisphere bovine production environments and possible future production locations. The greatest impacts of climate change will be in the low elevations in tropical and subtropical regions. Global regions above 30° and below 60° latitude with reliable rainfall will be least affected by current projected levels of climate change. This work provides the basis for computational animal design for guiding agricultural development via breeding programs, genetic engineering, management options including siting or the manipulation of other relevant environmental and animal variables.

## 1. Introduction

Climate spaces were first defined in an effort to understand how climate constrains the energetics of animals of different sizes, morphologies and with different physiological properties [1]. This early work only considered the survivability of the animals. Here, we extend the concept to include climate impacts on reproductive capability and find that there are not only climate spaces for daily function, but climate cliffs that represent reproductive failure in the face of climate warming.

Individual animals have “priorities” that are first for survival, then for growth and reproduction using discretionary mass and energy. Minimization of maintenance energy tends to maximize discretionary energy and mass available for locomotion, immune responses, growth or reproduction for any given local microenvironment. Thanks to the substantial amount of research available on domestic bovines (e.g., [2,3,4]), data are available to test a generic bovine model for computing milk production capacity based on local environments and animal properties. In this study, we use milk production as a surrogate of “reproductive output”.

A broadly tested (e.g., [5,6,7]) generic biophysical microclimate and animal heat and mass transfer model, Niche Mapper (NM), was adapted for use on bovines. This consisted of obtaining bovine-specific morphological, physiological, behavioral, heat and mass transfer, feed properties and local GPS coordinates to compute local hourly microclimates and bovine maintenance and milk production potential over a typical annual cycle. Tests of NM’s calculations against independent data sets [8] and experimental data from a commercial research farm show excellent agreement. Other tests of the model where experimental data are not available show results that are consistent with expectations (see below). The development of these mechanistic models opens the possibility of rapid quantitative determination of combinations of animal properties and environmental variables that will mitigate climate warming impacts on global milk production quantity and quality.

Background:

(a) Climate concerns. Heat stress is a subject of increasing concern for those in the dairy industry. Multiple publications, as shown below, show that dairy cows unable to eliminate excess body heat produce milk less efficiently and provide lower milk yields, possibly because of decreased rumination time with heat stress [9].

Heat stress results in reduced dairy farm profits, especially in hot and humid climates. In the United States, heat stress results in estimated annual losses ranging from USD 1.69 to 2.36 billion in the livestock industry alone. Within these losses, USD 897 to 1500 million occur in the dairy industry [10]. Farmers can mitigate these losses by manipulating the amount or quality of feed in terms of water or nutrient content or by upgrading their bovine cooling system. However, these options can often be costly and lead to greater economic losses than increased revenues [11]. These economic losses will impact U.S. farms with Holstein cows, especially in southern regions. Holstein cows are the predominant breed, representing 86 percent of all U.S. dairy cows [12]. 

Heat stress is likely to increase in the future, for humidity and temperatures are predicted to increase worldwide [13]. Between 2030 and 2052, the majority of GCMs project that global temperatures will increase by at least 1.5 °C [14]. Human activity is primarily responsible for this increase, causing about 1 °C of global warming above pre-industrial levels [15]. Climate scientists have predicted with high confidence that we will observe future increases in mean temperatures in land and ocean regions as well as increased hot extremes in the most populated areas [16]. Specifically, in the United States, the warmest daily maximum temperature is predicted to increase by 5 °C [17]. As temperatures increase worldwide, climate models have shown that relative humidity will stay approximately constant, contributing to an increase in specific humidity due to air’s increased capacity to hold more water vapor at higher temperatures [18]. This means that bovine evaporative water-cooling potential will be similar as climates warm.

(b) Model approaches and evaluations: Many correlative (regression model) studies focus on the impacts of climate variables, e.g., air temperature and humidity, on heat stress of lactating dairy cattle, e.g., [11,19,20,21,22]. Some of the impacts include increased water intake, reduced milk production and reduced forage quality and nutrient availability [23]. However, many knowledge gaps exist, e.g., haircoat (fur) properties, fecal water content, heat and mass transfer coefficients and capacity to respond to heat stress in warmer and wetter environments with larger body size. The effects of increasing temperatures on tropical and subtropical bovine species are not well studied. It is also uncertain how increased water intake may affect future water resources, as well as how these changes may impact livestock in many developing countries [23].

A comprehensive review of approaches to measuring and modeling heat stress in dairy cattle was recently published by Neves et al. [9]. They point out that direct animal measurements to identify heat stress (e.g., respiration rates or body temperatures) are limited, because they “identify the signs of heat stress and not the conditions that potentially lead to heat stress (pre-heat stress conditions), so it fails to prevent the deterioration of the animal’s health”. Thus, indices provide information on the level of discomfort. However, with DNM, knowing what the day’s or week’s weather will be, one can anticipate when heat stress will occur, which cows will be most affected and the level of reduction in milk production. 

Multiple indices that include not only temperature and humidity but also wind speeds and solar radiation have been developed to assess the thermal state of the heat-stressed animal. However, according to Neves et al., these were developed for different geographic locations and varied diets and farm systems [9]. The authors note that “for that reason, numerous studies comparing the performance of bioclimatic indexes under specific conditions have been drawing distinct (different) conclusions.” According to Ji et al. [24], “it is clear that the use of different bioclimatic indexes can lead to opposing conclusions: from neutral conditions, with one index, to moderate heat stress conditions, with another one, or even odd outcomes”. DNM uses no indices, only mechanistic equations. As shown below, different solar inputs or body sizes at the same humidity and temperature can result in very different levels of behavioral and physiological responses.

Neves et al. [9] point out that it is unrealistic to assume uniform thresholds for categorizing the level of heat stress of all animals on a farm, as the animals have varied biological attributes (age, phenotype/genotype and production level), and the animal’s thermal state is highly dependent on the specific environmental conditions each animal is exposed to. DNM captures all of this because of its embedded GPS-based microclimate model that defines the full range of available local microclimates on an hourly basis and is flexible in terms of animal properties, which allows it to identify individual animals that will or will not be affected by heat stress. 

In Neves et al.’s [9] review, in the section entitled “Prediction of Heat-Stressed Cows”, they state, “a robust tool able to predict the conditions in which the animal will face heat stress is the desired output. This will provide a non-invasive method to prevent the animal from being exposed to extreme conditions while allowing more efficient management of the resources (water/energy) to cool down the animal”. As we illustrate in this paper, this is exactly what mechanistic models like DNM do.

Finally, Neves et al. [9] state under “Mechanistic Models”, “However, only one asymmetrical environment condition option is considered (i.e., exposed area to shadow or direct sunlight) and, for that reason, it is not possible to analyze the effect of non-uniform distribution of environmental conditions on cow thermal state (e.g., it is not possible to simulate the heat transfer between the body region of a laying cow and the ground). To approximate realistic scenarios, it is necessary to determine proper boundary conditions by body region. Additionally, another relevant requirement is the reference values used to characterize the neutral conditions (e.g., temperature by body layers) according to the cow’s physical conditions (e.g., age, days in milk)”. DNM addresses all these issues. 

(c) Unique properties and advantages of DNM: One of the major shortcomings of other heat transfer models of cows is that none of the others have microclimate models associated with them, so that if a cow is modeled to lie down instead of standing up to avoid heat stress, adjusting wind, humidity and local air temperature accordingly is not part of the modeling process of other models. DNM has an array of user-specified allowable automatic options if the current hour’s environment could be imposing either heat or cold stress. It automatically explores user-authorized mechanisms for minimizing heat or cold stress. DNM incorporates morphological (postural) changes and knows the microclimate differences between standing and lying down, as demonstrated in [7,25]. The behavioral options in DNM allow for modeling behavioral choices, like shade seeking, if the model detects that the animal would be in heat stress by remaining in the sun. Physiological options in DNM allow for changes in blood flow to the skin or panting or sweating to be implemented to help mitigate heat stress. In short, it is the incorporation of morphological, physiological and behavioral adjustments in the context of local available microclimates that can occur for each hour of simulation as well as the capacity to simulate a cow outdoors or indoors in virtually any environment the user specifies that is part of what makes DNM unique.

In summary, DNM’s unique approach includes: (1) a generic, broadly tested microclimate model to determine the hourly range of local microclimates that affect cow energetics and behavior indoors or outdoors for any geographic location; (2) a distributed heat energy generation equation that ties temperature to heat flux in the animal and accounts for simultaneous distributed internal heat generation and conduction rather than using a point source of heat and conduction from core to skin [26]; (3) coupled heat and mass transfer equations for each body part (head, neck, torso, front, back legs and tail) of the animal that allow for differences in heat fluxes from different parts of the animal morphology; (4) an integrated porous media model for haircoat for each part of the animal; (5) a validated 3D reconstruction of the morphology of the cow that assures accurate surface areas and volumes; (6) heat and mass transfer coefficients that scale isometrically that allow for any body size changes; (7) physiological and behavioral feedback adjustments to local environments to minimize maintenance requirements, maximize comfort and achieve production targets; (8) feed properties as they affect heat and mass balances and feed requirements; (9) a record of global-scale tests of the underlying microclimate and animal models for both climate and species, representing all the major classes of vertebrates; (10) integration of the heat, mass, molar and momentum balances that provide interacting constraints on the numerical solutions for response variables of interest, e.g., milk production; (11) operational capability for temporal (hourly) and spatial (GPS) resolution of simulations.

Objectives: Our first objective is to quantify some of the knowledge gaps through biophysical-bioinformatics modeling and specifically calculate how predicted climate changes in different regions of the Americas and Asia will affect future bovine milk production and water and feed needs for outdoor (grazing) and indoor (confinement) dairy production systems. Our second objective is to investigate how morphological and physiological differences affect these dependent variables. 

“Metabolic chamber” steady-state tests of DNM were performed using data from the literature and our own data. Global locations simulating monthly local weather at each site over a year used a Yates’ algorithm to define main effects and interactions of location, body size and diet type on milk production potential for dairy cows for current weather and future warming scenarios in the northern hemisphere. Finally, we analyzed grazing shade availability and fecal water content for their impact on milk production potential.

Our hypotheses are as follows: (1) Milk production and feed and water needs will be most affected by the latitude, elevation and size of the cow and least affected by other cow physical characteristics. (2) Higher temperatures and humidity will exacerbate animal heat stress, resulting in lower milk yields, greater water intake and altered feed needs. (3) Greater variance in local climates may have sudden, strong effects on large cows’ milk production.

## 2. Materials and Methods

### 2.1. The Niche Mapper Computer Model

Climate change effects on bovine species at low- (12°), medium- (30°) and high-latitude (60°) locations at ~30 m elevations in the Americas and in Asia (Figure S1) were computed using DNM software (Version 5) derived from a generic microclimate-animal model, Niche Mapper™. Niche Mapper is a broadly tested set of integrated bio-engineering software. The program uses a combination of heat and mass transfer equations first to establish local hourly microclimates. Those microclimate data for the hottest and coolest local environments, coupled with morphological and physiological properties and behavioral characteristics of an animal, are then used to compute hourly heat and mass exchanges between the animal and its environment [1,26]. 

Niche Mapper is composed of two sub-models: a mechanistic microclimate model and a bioenergetics-biophysics animal model. The microclimate model uses macroclimate data including air temperature, wind speed and relative humidity obtained from airports, cloud cover from satellite data and geographic coordinates that provide information for the embedded generic solar radiation model as inputs. It uses heat and mass balances similar to those described below for an animal to calculate above- and below-ground hourly local environmental conditions, such as profiles of air temperature, wind speed and relative humidity for 2 m above ground to the ground surface, solar and thermal radiation from the sky and ground (Figure S2) and temperature profiles down to 2 m deep into the soil.

The bioenergetics-biophysical animal model utilizes hourly microclimate output from the microclimate model with animal heat and mass transfer properties derived from Computational Fluid Dynamics, CFD, e.g., as in [27], as well as other morphological, behavioral and physiological properties [28] to determine the demands of mass and energy of the animal and the feasible range of behaviors and physiological adjustments that accommodate those demands for the local hour’s environment (see Supplementary Materials).

Niche Mapper calculates metabolic rates that maintain core temperature and physiological functions based in part on the heat balance equation:

*Q_in_* + *Q_gen_* = *Q_out_* + *Q_stored_*. (1)

In this equation, the heat received by the animal from the environment, *Q_in_*, plus the endogenous distributed metabolic heat production, (*Q_gen_*) [26], which includes heat of fermentation and milk production, must equal the net heat loss to the local microenvironment, *Q_out_*, plus any heat stored in the body via body temperature change, *Q_stored_*, to maintain a core temperature within a user-specified range, as described in the supplementary material in [28]. The heat in, *Q_in_*, consists of the sum of solar radiation absorbed, *Q_sol_*, and thermal radiation from the sky and ground, *Q_IRin_*. The heat out, *Q_out_*, consists of the sum of heat fluxes due to convection, *Q_conv_*, conduction, *Q_cond_*, evaporation, *Q_evap_*, and thermal radiation to the environment, *Q_IRout_* (Figure S2).

Each of these heat flux terms has its own mechanism equation [28]. If the animal has fur or feathers, a porous media model for fur or feathers computes the heat transfer from the skin to the fur–air interface via conduction and radiation terms, as described in the supplementary material in [28]. To gauge how well the animal is tolerating a particular climate, the calculated metabolic rate is compared to a target resting or active metabolic rate. These two rates represent the metabolic range of the animal in the current climate and activity level. If the calculated rate falls below the resting rate, the animal must take action to avoid dying of hyperthermia. If the calculated rate is above +5% of the target active rate [25], then the animal must take action to avoid hypothermia. A calculated rate between the resting and active rate indicates that the animal is not heat stressed and can maintain its normal active level of endogenous heat production (including heat as a byproduct from milk production). If the calculated rate is outside the resting–active range, a series of behavioral and physiological responses are triggered, such as seeking shade or shelter, changing posture, piloerection, sweating, panting and changing blood flow rate to the skin, as described in [28]. 

Additional equations that NM uses are mass balance equations of the form:

*M_in_* = *M_ox_* + *M_out_* + *M_stored_* (2)

**Figure 1:**
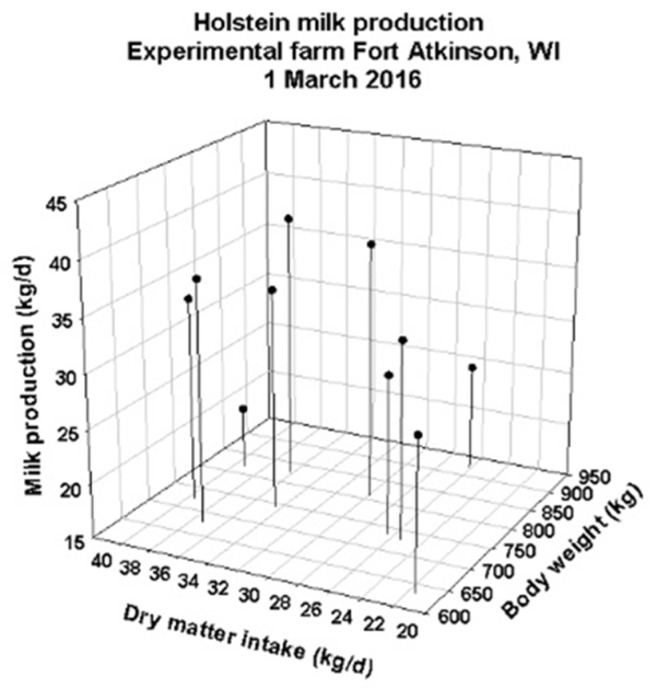
Experimental data on 10 individual cows’ day’s milk production and dry mass feed intake on a Wisconsin commercial experimental dairy farm’s enclosed barn equipped with fans for cross-ventilation control.

**Figure 2:**
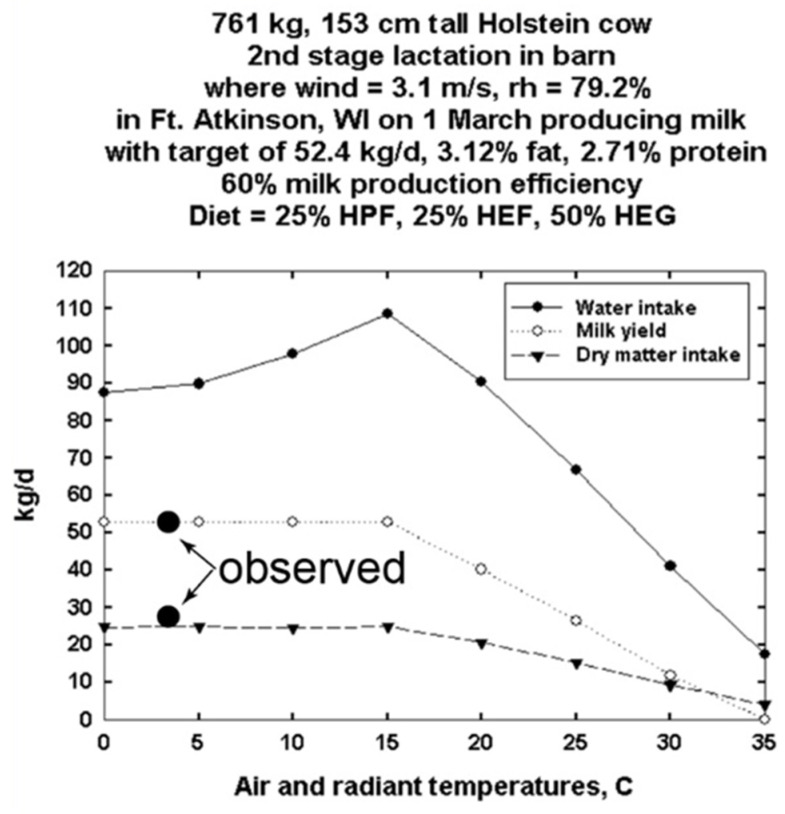
Dairy Niche Mapper validation from the Wisconsin commercial experimental farm data. Calculated vs. measured dry matter feed intake and milk production are for the “average” cow representing the group of 10 cows in Figure 1. Other temperatures were also computed to illustrate expected feed and water intake and milk production potential for the average of the measured milk properties and diet type used on the measurement day.

**Figure 3:**
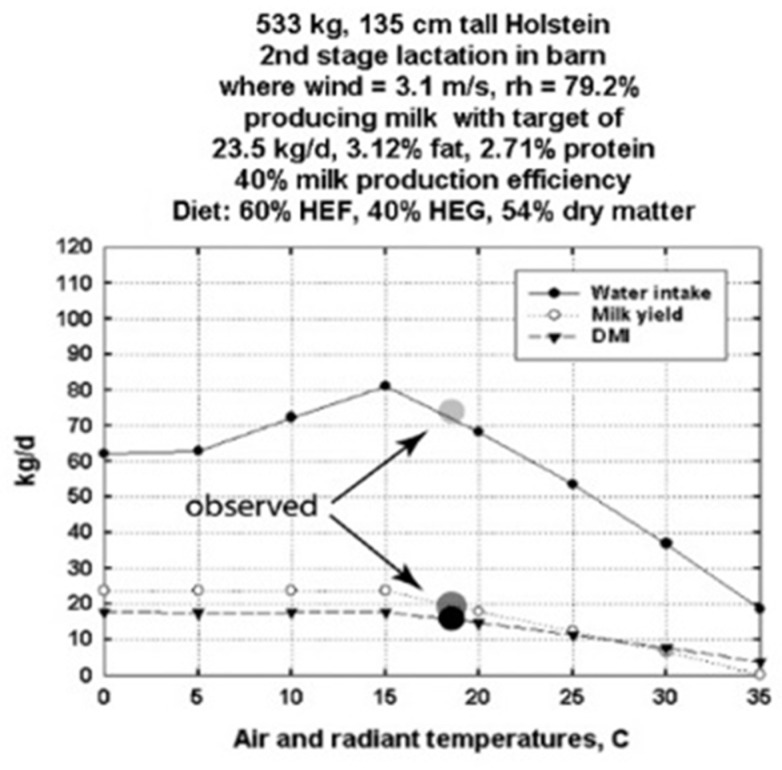
Dairy Niche Mapper validation using literature data from Table 1 in Appuhamy et al. (2016) [8]. Calculated versus measured dry matter feed and water intake and milk production are for the “average” cow, representing available data in the literature in which environmental temperatures averaged 18.1 °C. Wind speed and humidity were not measured in the cited experiments, so the same values for wind speed and humidity as the Wisconsin farm experimental conditions were used. Temperatures from 0 to 35 °C were used to illustrate expected food and water intake and milk production potential for these average cows’ measured milk production and diet.

There are three mass balance equations, one each for feed, water and air. They are coupled with each other and with the heat balance equation. In the case of feed, *M_in_* represents the amount and characteristics of feed and the amount of water consumed by the animal. *M_ox_* represents the mass that is oxidized to do chemical and mechanical work. *M_ox_* is calculated using the thermodynamic inefficiencies of chemical and mechanical work that result in heat generation. *M_out_* represents the mass leaving the animal from various body systems (e.g., the excretory, respiratory and reproductive systems). *M_stored_* represents the amount of mass stored in various biological forms, e.g., flesh, bone and fat. Since the oxidation of mass (*M_ox_*) releases heat energy (*Q_gen_*), the heat and mass balance equations are coupled (Figure 2 in [26]). Niche Mapper combines and solves those equations numerically to calculate bioenergetic outputs (Figure 1, Figure 2 and Figure 3). These generic heat and mass balance equations hold true regardless of the biological scale the user is considering.

### 2.2. The Dairy Niche Mapper Model

The Dairy Niche Mapper (DNM) program expands the NM program by adding lactation costs in terms of mass and energy needs for different target levels of milk production, milk protein and fat content and milk production efficiencies. It also includes six additional modifications: (a) cow 3D morphologies (Figure S2), (b) heat and mass transfer coefficients specific to cows, (c) a range of cow haircoat reflectivities based on user-specified color percentages of black, white, brown, red or tan (cow haircoat reflectivities were measured with an ASD fieldspec portable spectroradiometer, with a spectral range of 350–2500 nm and a spectral resolution of 3 nm from 350–1100 nm, 10 nm from 1101–2500 nm), (d) a full complement of NRC (2001) diet composition data (see below) that the user selects, (e) a cap on thermoregulatory cutaneous evaporative water loss of 400 g/m^2^-h based on measurements of a broad range of cow breeds [29] and (f) a gamma function milk production curve proposed by Wood [30]: yield = *at^b^e^ct^*, where *t* is time after calving, and *a*, *b* and *c* are constants describing the curve for a particular herd. Generic constant values from Whittemore [31] were used, and a 305-day milking period [31] was assumed. 

Thermodynamic milk production efficiency is the biochemical efficiency of converting feed chemical energy to the stored chemical energy in milk. This definition is related to the definition of feed efficiency, i.e., energy-corrected milk (ECM)/dry matter intake (DMI). The calculation of ECM standardizes actual milk production to 3.5% milk fat and 3.2% milk protein using the formula ECM (kilograms) = (0.3246 × kilograms of milk) + (12.86 × kilograms of milk fat) + (7.04 × kilograms of milk protein) [32]. Feed efficiency for the second lactation typically varies between 1.19 and 2.01 [33].

Metabolic rate in hot conditions is balanced in DNM, if possible, by allowing greater cutaneous and respiratory evaporative water loss up to known physiological limits and at each hour’s available air temperatures, wind speeds, relative humidities and solar and thermal radiation. 

Dairy Niche Mapper’s graphical user interface (GUI) allows novice users to select any global location from an interactive global map and specify a variety of independent variables such as cow weight and height, haircoat properties, e.g., hair color, pelt depth and hair length. When considering the morphologic inputs (e.g., height, weight, haircoat depth and hair length) for a Holstein cow (Table 1), we used data from the National Dairy Heifer Evaluation project [34] and measured values from 10 cows from PHD R&D (Fort Atkinson, WI, USA), an independent research farm that provided environmental and animal data used to validate the DNM model. The research farm follows the Guide for the Care and Use of Agricultural Animals in Research and Teaching, 4th ed. [35]. The average percent milk fat (3.2%) and protein (2.7%) from the 10 experimental farm cows was used in the calculations. An average fecal water content of 80% was measured from the 10 cows at this commercial research farm. There are a few studies that show slight differences in Holstein production and physiology depending on the percentage of white versus black haircoat color [36,37]. We consistently selected a 50:50 white-to-black color coat ratio to avoid color variation in our global study to focus on the locational differences and their effects. 

The DNM calculations of milk production potential for countries outside the United States implicitly assume that feed availability and quality, as well as all the other supporting infrastructure for dairying as practiced in the United States, are present in all countries. In fact, many of our estimates for milk production are much higher than existing values due to lack of feed quality and availability, management practices, breed of the cattle and other variables. We decided to take an “average cow” approach based on US cow capabilities for two reasons. The first is that many developing countries in the world are moving to increase their production capabilities by addressing the problems they face in terms of the genetics of their animals, management practices and a stable supply of appropriate feedstocks, especially for dry season-wet season environments. As these countries achieve their goals, they need to be able to anticipate how climate change may act to subvert some of those expectations.

Computing local microclimates: Geographic location selection used New et al.’s [38] average monthly maximum and minimum macroclimate data (air temperatures, wind speeds, humidity, cloud cover) for the selected location, and it computes hourly solar loads from the time of day and year, latitude, longitude and elevation. Thermal radiation from the sky is computed from regressions of air temperature at 2 m [39], and ground thermal radiation is computed from the microclimate model’s hourly computation of the heat balance on the ground surface. The user can also specify local environment conditions, e.g., shade availability, the presence or absence of a barn or other shelters, diet composition, e.g., forage and grain types and percent of each feed type, time of calving, milk composition, e.g., lipid and protein content, and current and future temperature predictions. Once the necessary variables are entered, e.g., as in Table 1, the user can observe graphically and in tabular form how the independent variables affect the dependent variables, such as current and future milk production, water intake, forage needs and grain usage.

Diet types: There are six different diet types (Table S3) that the user can specify in the DNM program: high-protein grain (HPG), high-energy grain (HEG), medium-protein and grain (MPG), high-protein forage (HPF) and high-energy forage (HEF). The approximate composition of each diet type has been generated from data in the Nutrient Requirements of Dairy Cattle, Table 15-1, p. 283 in NRC (2001), (https://www.nap.edu/catalog/dairymodel) (accessed on 22 November 2022).

The content of dry matter, protein, fat, fiber and starch differs between diet types and may affect the animal’s overall feed and water needs depending on other variables (i.e., location, climate, target maximum daily milk production, month of calving, month of the annual lactation cycle and feasible overall metabolic rate, consistent with the body temperature to be maintained). 

### 2.3. Niche Mapper Validations

Diverse methods were used to validate the generic models in NM, the basis for DNM, in the laboratory and in diverse environments. Some of these include validations for amphibians, e.g., reptiles, e.g., [40,41] and birds. There are also multiple tests using wild mammals. For example, we have used deer mice [26], pikas [42], koalas, polar bears [28], pandas, elk, free-ranging vervet monkeys [25], humans [5] and the Holocene woolly mammoth [43]. We have also validated NM for ruminants like the Japanese Serow deer [44], elk and moose [7].

Early experiments on dairy cattle by Berman [2,45] and by Gebremedhin and colleagues from 1981 through 2015 (e.g., [29]) provide a rich background for bovine model development. Our modifications of NM and the incorporation of our microclimate model to characterize local microenvironments allowed us to determine environmental constraints on bovine feed and water consumption needs for milk production potential at local and global scales for indoor (confinement) and outdoor (grazing) environments. Once DNM was validated from empirical data in the literature, sensitivity analyses were conducted for cow sizes that represented our data and other experimental groups’ data. Finally, sensitivity analyses were performed over the size range of present-day dairy cows to determine current and future environmental effects on food and water needs, metabolic rate and milk production potential.

### 2.4. Validation Methods for Dairy Niche Mapper (DNM)C

Two independent methods were used to test DNM estimates of metabolic rate, milk production and feed and water intake accuracy. The first method was to collaborate on a study of 10 cows at a local independent research farm that has their own approved protocols for dairy cow research. The farm staff collected all data following the current best practices recommended for research and teaching [35]. Our study simply monitored standard practices, such as a day’s feed consumption, milk yield and quality and feed composition, as well as indoor and outdoor microclimate measurements and cow morphometrics. Some of the data collected were used as input to the cow model part of DNM, e.g., body weight, shoulder height, haircoat depth, hair length and fecal water content. Microclimate data, i.e., air temperature, wind speed and relative humidity measured in the barn and outside the barn drove the microclimate model part of DNM. Calculated feed intake and milk production were compared against measured data. Feed intake and milk production data were collected at the barn’s temperature of 3 °C, a wind speed of 3.1 m/s and a relative humidity of 79.2% in the month of March. 

The second validation method was to use the literature data in [8] to compute feed and water intake and milk production based on an average cow size, ambient temperature and the milk production reported in the subset of their data in Table 1, where temperatures are reported. We assumed wind speed and relative humidity were the same as that measured in the Wisconsin barn, since those data were not reported for the literature-based data, although since the study occurred in Davis, California, the relative humidity may have been lower.

A third validation not reported here that only applies to metabolic rate and not milk production was the use of our own data in the literature to directly compare metabolic measurements on Holstein calves versus calculated values for metabolic rate [3,26] that showed close agreement between measurements and model results from 5–15 °C. 

These comparisons, though limited to cows and calves, support the broad applicability across species and size ranges for the generic microclimate and animal models in Niche Mapper.

### 2.5. Metabolic Chamber Simulations

Metabolic chamber simulations are useful for estimating the impacts of short-term climate extremes that constrain reproduction and survival, because they provide well-defined environments for evaluating animals and climate effects on their physiology. For all the example simulations of metabolic chambers that follow, we simulate a 643 kg Holstein cow based on data from Chen et al. [46] and Wang et al. [47]. We proceed from the “standard” 2D graph of metabolic rate as a function of air and radiant temperature, solar radiation, relative humidity and wind speed fixed for the simulation to 3D representations using temperature and wind speed as independent environmental variables and response variables metabolic rate, milk production potential, food requirements and water requirements for a 100% black healthy Holstein. Additional 3D graphs for 100% white Holsteins with and without solar radiation and different levels of relative humidity may be found in the Supplemental Materials (Figures S2–S7a–d). Contour graphs or “top views”, which reduce the three dimensions to two dimensions, are presented in the Supplemental Materials to help quantify the response variables explicitly in Figures S6a–S7d. 

To help compare the DNM approach to a more familiar temperature-humidity index, THI, results are also presented in temperature vs. relative humidity axes at different wind speeds and solar radiation loads to visualize environmental spaces and cliffs for a target 50 kg/d of milk production (Figure S8a–d). 

“Temperature-humidity milk index” plots show the milk production potential for a black Holstein without solar radiation at 0.1 m/s wind speed (Figure S8a) versus 3.0 m/s (Figure S8b). The edge of the “cliff” moves to the right, i.e., higher temperatures are tolerated at 3 m/s, but the cliff is steeper, as indicated by the smaller space between the contours in Figure S8b.

Applying 500 W/m^2^ of incident solar radiation onto the 643 kg black Holstein in Figure S8b generates Figure S8c and illustrates how milk production contours move back to the left toward lower tolerated temperatures. Slight changes in wind speed (3.0 m/s versus 2.8 m/s) can collapse milk production precipitously (Figure S8d), showing that only a maximum relative humidity of 10% could be tolerated, and the amount of milk that could be produced collapses to only 10 kg/day in the most favorable available conditions. Calculations for a 2.9 m/s wind speed shows virtually the same contour lines as those at 3 m/s. Thus, a change in wind speed of only 0.1 m/s at a crucial combination of environmental variables could shut down milk production completely. The reason for this apparently bizarre behavior is seen in Figure S8e. Here, we see that by lowering the incoming solar radiation by 20 W/m^2^, we drop to a fifth unseen solar radiation dimension to find a plane of production of 50 kg per day at the same 2.8 m/s wind speed that at 500 W/m^2^ was not attainable.

### 2.6. Climate Effects on Cows in Confinement

Cows in barns are in environments that are intermediate between metabolic chambers and outdoor conditions. The radiation environments, both solar and thermal, are more “muted” and uniform in shelters. Validation of DNM by way of our commissioned confinement experiment and data from the literature provided confidence that we could utilize DNM to explore confinement environments and how they might modify cow input needs and output capabilities in the context of temperature, humidity, wind speed, solar and thermal radiation and body size from the three experimental groups, i.e., the Wisconsin barn experiment and the literature data summarized by [8] and the early calf experiments [3,26]. Based on the validation data results, we decided to first compute “indoor” weather effects on the smaller average size cow (533 kg) represented by the data in Appuhamy et al. (2016)’s data set, to determine on average how indoor environmental conditions would affect these cows’ water and feed needs and milk production across the temperature range from 0 to 35 °C. Then, we compared those results with the larger size cows (761 kg) from our experimental barn data for the same range of environmental conditions at low and high wind, and we finally expanded the simulations’ scope to six spatially explicit average monthly outdoor climate sets and cows across the maximum cow size range in the literature, i.e., 364 kg–1273 kg, processing the results in Yates algorithm calculations.

### 2.7. Experimental Design and Sensitivity Analyses for Global Locations, Body Size and Diet Effects on Milk Production Using the Yates Algorithm [48,49]

In the outdoor (grazing) sensitivity analysis study, we first tested three key independent variables: latitude (12 °N vs. 60 °N), cow size (i.e., size of a Jersey cow at 364 kg vs. a large Holstein at 818 kg) and diet (high-energy grain vs. high-energy forage) using a Yates algorithm analysis [49]. These 3 primary variables were identified using Yates from a larger suite of possible variables. We compared the effect of each of the three independent variables on the response variables of cow milk production, water and feed needs. Site elevations were approximately constant at 21–36 m for all six sites (Table S1). We assumed for comparison purposes that all the cows were Holsteins, even though in tropical lowlands, crosses between *Bos taurus* and *Bos indicus* are common, resulting in smaller cows with higher heat tolerance and disease resistance and less milk production. We assumed that the 50% black, 50% white cows were outdoors but could choose shade up to 100%. Specific details about the input variables used are in Table 1 and Table 2. We selected low, middle and high values of the three independent variables based on current dairy latitudes and the range of common cow sizes and diet types. All simulations used the respective local monthly climates for each site. For each independent variable, e.g., for latitude, we set the low value at 12°, the middle value at 30° and the high value at 60° north latitude. 

Our center replicate conditions were the means for each variable’s range. Using a full factorial design in the three independent variables, we computed main effects and interactions on the dependent variable responses. Combinations of higher-order interactions (typically small effects) were not confounded with lower-order ones (typically larger effects) for our full factorial designs, as defined in Box et al. (1978). The center replicate condition allows for the determination of linear vs. non-linear responses to combinations of the independent variables and reduces replicate needs on the “corners” of the design. Center replicates in Yates’ algorithm [49] also provide a frame of reference for any changes that might occur when fractional factorial designs must be run sequentially through time to recover a full factorial design. We computed how much each independent variable affects each dependent variable of interest (e.g., milk production, water and feed needs). We show how the combined interactions between two or three independent variables affect the dependent variables, and whether the combined interactions have a greater or lesser impact compared to an individual independent variable. Independent variables such as temperature change that have the greatest effects can be identified for cows with differing physical, biological and locational characteristics. All other input variables in Table 1 and Table 2 were held constant for this analysis.

### 2.8. Global Simulations for Dairy Animals Outdoors

Table S2 summarizes the annual monthly climate data [38] used to compute hourly values outdoors at each of the six selected locations in Nicaragua, the USA and Canada in the Americas and in the Philippines, China and Russia in Asia. The experimental design for cows in Table 2 specifies the bovine sizes and diet types at each of the six localities that would be run to obtain annual summaries of water and feed needs and milk production potential. Table S4 shows the calculated feed need changes on the assumed climate change for Holsteins.

All the global simulations are for free-ranging grazing dairy cows. Simulations for the effect of shade on heat or cold stress were considered by triggering the shade available option in the DNM GUI interface. The microclimate model calculates the hottest and coolest available microclimates for each hour of the average day for each month of the year. DNM automatically assumes a range from 0% to 100% shade availability for the free-ranging grazing cows during each day in those calculations. We used the GUI behavioral option for the cow for whether shade is available, so it can select the appropriate level of shade in the cooler shaded microclimate when hot or the warmer daytime unshaded microclimate when it is cold. 

The GUI interface was also used to determine the effect of the month of calving on annual milk production. We looked at representative dates for January, March, July and September. We looked at both annual and monthly milk output. 

## 3. Results

### 3.1. DNM Validations Using Data from an Experimental Dairy Barn

Even though Niche Mapper has been extensively tested in a broad range of habitats and species, the modifications to create Dairy Niche Mapper must still be tested for accuracy. Figure 1 presents the observed data on milk production versus dry matter intake and body weight of the ten cows measured in an experimental barn near Fort Atkinson, WI. Because of the natural variation in feed intake and milk production among individual cows of different sizes, we elected to do calculations for the “average” cow based on the averages of these data. 

Figure 2 shows the DNM computation of water intake, milk production and dry matter intake from 0 to 35 °C for the average cow, 761 kg, 153 cm tall, in the group of 10 cows. Figure 2 shows calculated milk production for barn temperatures from 0 to 35 °C at the wind speed and humidity measured in the experimental barn. The experimental measurements of milk production and feed intake were made at 3 °C, the environmental conditions for that day. Although the calculated results are plotted for a broad temperature range of 0–35 °C, the calculated milk production and feed intake at 3 °C agree well with measured values. We were not able to obtain water consumption data, so there was no “measured” water intake datum for 3 °C, although we calculated what it would be to balance the heat and mass transfer equations. As mentioned above, thermoregulatory cutaneous water loss was not allowed to exceed 400 g/m^2^-h [29]. All 10 cows were assumed to be in their second lactation. 

### 3.2. DNM Validation Using Peer-Reviewed Literature Data 

Figure 3 shows the computation of water intake, milk production and dry matter intake from 0 to 35 °C for the average cow in the data, containing ambient temperature reported by Appuhamy et al. (2016) in their Table 1. At 533 kg, their average cow was smaller, and we estimated a 135 cm tall cow, which allowed us to isometrically downsize our 3D cow and compute its heat transfer properties using CFD, e.g., as in [27]. The experimental observations for milk production and feed intake are plotted at 18 °C, the average temperature Appuhamy et al. reported, along the 0–35 °C temperature scale. In this case, water consumption was also reported so we could compare our calculated values with observed values. Experimental assumptions are listed in the figure legend. The “target” maximum daily milk production used in these calculations was the observed average value in the data subset section of Appuhamy et al.’s Table 1 of 23.5 kg per day.

### 3.3. Metabolic Chamber Simulations

**Figure 4:**
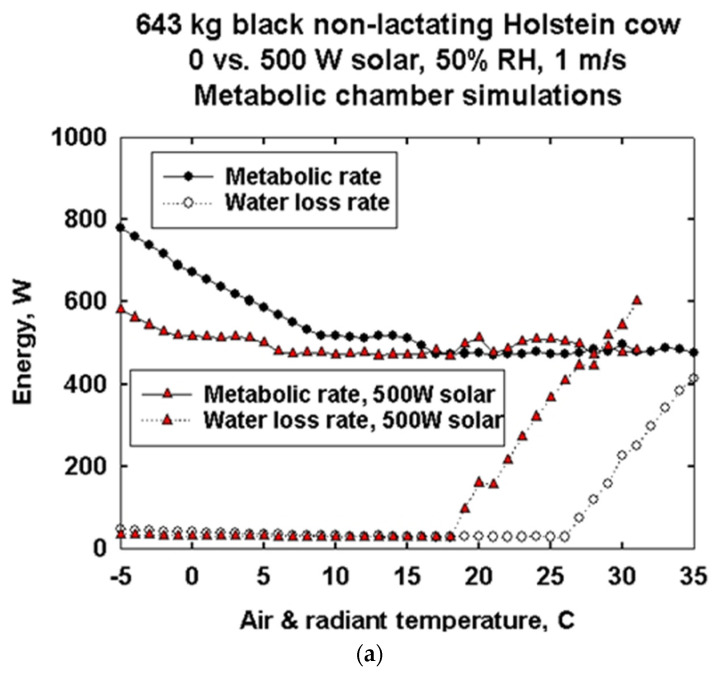

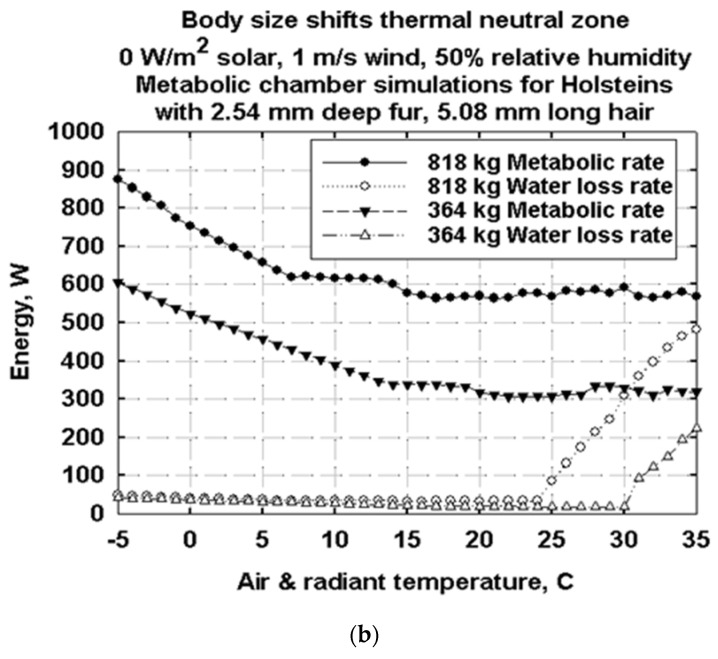
“Standard” 2D representation of metabolic chamber simulations of a 643 kg black Holstein nonlactating cow’s air- and radiant temperature-dependent metabolic rate with no solar radiation and with 500 W/m^2^ incident solar (**a**), each at 50% relative humidity and 1 m/s wind speed (**b**). Animals are assumed to be standing at all temperatures.

Figure 4a is a typical 2D plot of calculated metabolic rate and water loss as a function of environmental temperatures for a nonlactating 643 kg Holstein. The curves for no solar and for 500 W/m^2^ of solar radiation on the cow show a broad thermal neutral range from about 35 °C down to approximately 17 °C in no sun. Below 17 °C, metabolic rate begins to increase to maintain core temperature. For the same cow in 500 W/m^2^ solar radiation, metabolic rate increase at lower temperatures is delayed down to approximately 6 °C. For the simulations, we assumed that the core temperature is maintained at plus or minus an average of 39 °C, with a high of 39.4 and a low of 37.5 °C allowable. The rising water loss rates with and without solar radiation show when the cow must start evaporating water for thermoregulation. The initiation of thermoregulatory water loss occurs at ~18 °C with 500 W/m^2^ solar vs. ~26 °C when there is no solar radiation load. 

Figure 4b shows that body size influences the thermal neutral range. Here, we have simulated the typical smallest and largest size of Holsteins, giving them both the same fur properties. The small 364 kg Holstein has a thermal neutral range of approximately 14–30 °C. The large 818 kg Holstein’s thermal neutral range is approximately 7–24 °C. The thermal neutral range according to the seventh NRC Nutrient Requirements of Dairy Cattle is 5–20 °C.

**Figure 5:**
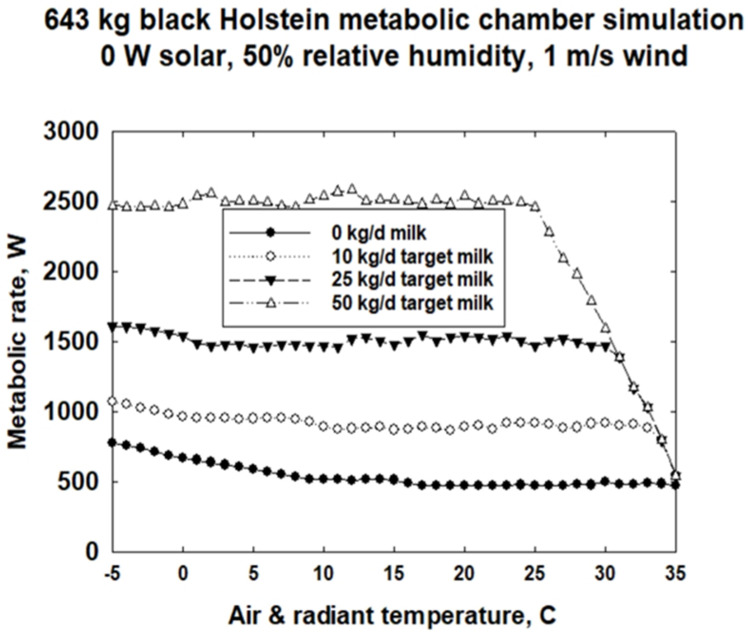
2D representation of metabolic chamber simulations of 643 kg black Holstein cows with different milk production targets for air- and radiant temperature-dependent metabolic rates assuming no solar radiation, 50% relative humidity in a 1 m/s wind.

The metabolic consequences of adding different lactation targets are substantial (Figure 5). First, milk production metabolic requirements can dwarf the main effects and interactions in the metabolic rate of a nonlactating cow. Second, the capacity to meet the milk target diminishes from 50 kg per day at 25 °C to no milk production at 35 °C for an environment with no solar, 50% relative humidity and 1 m/s wind speed. For the simulations, we set targets of 10, 25 and 50 kg/day of milk production.

**Figure 6:**
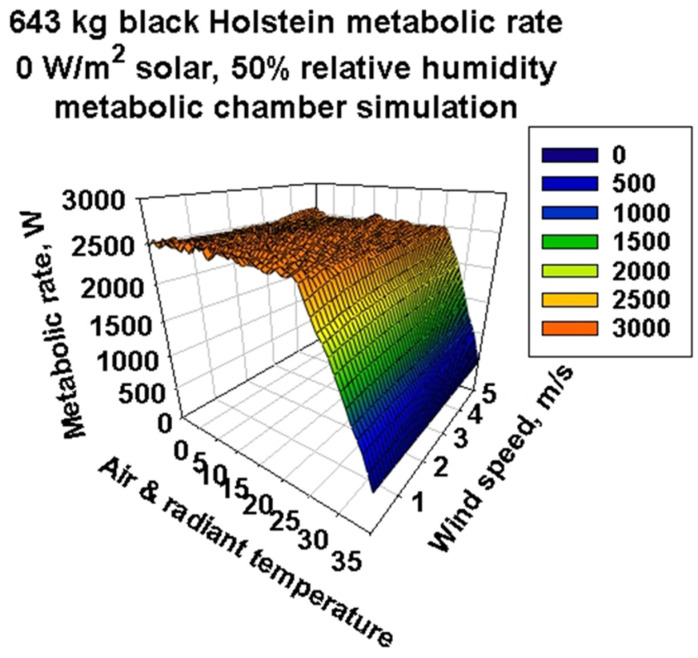
Three-dimensional representation of metabolic chamber simulations of a 643 kg black Holstein’s metabolic rate for air and radiant temperatures of −5–35 °C, wind speeds from 0.1 to 5 m/s and a single relative humidity of 50%. The horizontal flat plane at approximately 2500 W has some roughness in it due to various physiological and behavioral adjustments the model cow would make in response to the environmental constraints and a milk production target of 50 kg/day.

Figure 6 is a 3D metabolic response surface that has wind speeds from 0 to 5 m/s in 0.1 m/s increments. Now, we can see the climate space where the 50 kg/d milk target can be achieved. A “plain” of the feasible climate variable combinations lies on top, where the maximum metabolic rate is thermodynamically possible. The climate “cliff” to the right at higher temperatures shows rapidly declining feasible metabolic rates. The surfaces are smooth, but there are subtle curvatures near the top edge of the cliff that are not apparent in this view. The roughness of the surface reflects physiological and behavioral adjustments to maintain body temperature within the acceptable range.

**Figure 7:**
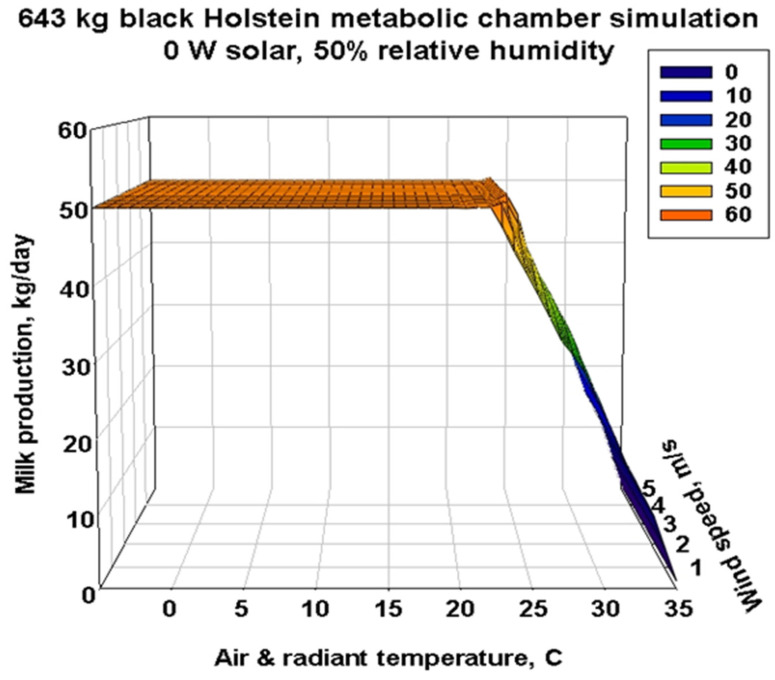
Three-dimensional representation of metabolic chamber simulations of the milk production capacity of a 643 kg black Holstein for air and radiant temperatures of −5–35 °C, wind speeds from 0.1 to 5 m/s and a single relative humidity of 50%. The horizontal flat plain at 50 kg per day milk production can be maintained until a temperature of approximately 23 °C at the lowest wind speed of 0.1 m/s. As wind speeds increase, approximately one more degree centigrade is added to the edge of the production cliff before it slides to 0 kg per day at 35 °C.

Figure 7 represents the milk production potential for the highest target milk production of 50 kg/day. The 3D plain surface where the target production can be achieved has a cliff edge at approximately 23 °C depending on the environmental conditions and animal properties shown here for this size of Holstein. 

The 3D response surface for food requirements, Figure S3, has the same 3D shape as milk production but different values on its vertical axis, food needs, instead of milk production.

**Figure 8:**
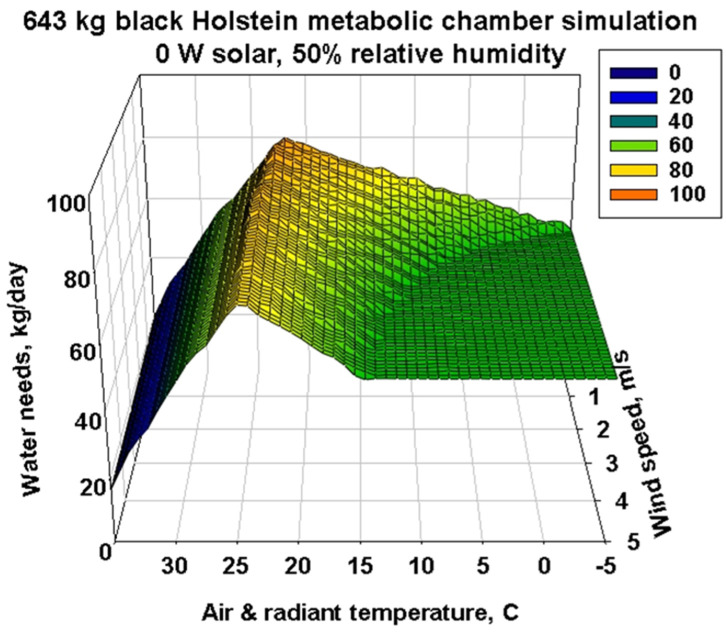
Three-dimensional representation of metabolic chamber simulations of water needs of a 643 kg black Holstein for air and radiant temperatures of −5–35 °C, wind speeds from 0.1 to 5 m/s and a single relative humidity of 50%. The x-y plane has been rotated 180 ° from the prior 3D response surfaces of metabolic rate and milk production potential to illustrate the complex relationships between the environmental variables and water loss rates of the simulated cow. The flat plane of low water loss requirements is in the near right hand corner of low temperatures and high wind speeds. Evaporation losses from the cow begin to increase on a curvilinear line at 5 m/s and approximately 15 °C. The initiation of escalated water loss is depicted by the edge of the plane curving around to the right and ending close to 0.1 m/s wind and −5 °C temperature. The escalating plane of increased water loss continues until approximately 22 °C at a wind speed of 5 m/s, where the “ridge” or edge of the cliff representing increasing heat stress curves around to about 22 °C at the lowest wind speed of 0.1 m/s. The downward slope to the left of the ridge as temperatures rise reflects the requisite metabolic depression that would occur given the heat generation of the animal due to the desired target milk production level of 50 kg per day and the environmental conditions. For more details, see Figure S6e,f.

The 3D response surface for water needs is much more interesting and complex (Figure 8). It has the same simulated environmental conditions as in the previous 3D figures. Figure 8 has been rotated 180° from the prior 3D surfaces to show how the transition from a steady low evaporative water loss at cold temperatures and high wind speeds rises to a peak water loss rate at approximately 25 °C. The ridge peak is slightly curvilinear with diminishing wind speed, bending at the lowest wind speed of 0.1 m/s to the coolest peak temperature of approximately 22 °C. To the left of the ridge is the “cliff”, where the water needs response surface drops rapidly because the metabolism must decrease due to increasing temperatures and diminishing wind speeds. The ridge defines the point at which the thermoregulatory functions are at a maximum and start to fail. The degree of curvature of the ridge and the rise on the far edge at the lowest wind speed are greater for the black Holstein than for a white Holstein with the same properties. This can be seen in the Supplementary Materials (Figures S3–S5), where the simulations of water loss at different amounts of solar radiation and higher relative humidity (greater heat stress) are shown. At the lowest wind speeds, the far edge (lowest wind speed) of the water loss surface curls upward as winds diminish, and the peak of the ridge bends more to a lower temperature at the lowest wind edge. One can see the effect on water requirements of black versus white color with 0 vs. 500 W/m^2^ solar radiation and 50% relative humidity (Figures S3, S4 and S6a–d). Contour plots of Figures S3 and S4 in Figure S6e,f allow one to more explicitly examine the quantitative water requirements due to coat color differences in sunlight at 50% relative humidity.

The contour plots for milk production (Figure S6a–d) likewise allow for more quantitative understanding of solar radiation and relative humidity impacts on milk production potential.

**Figure 9:**
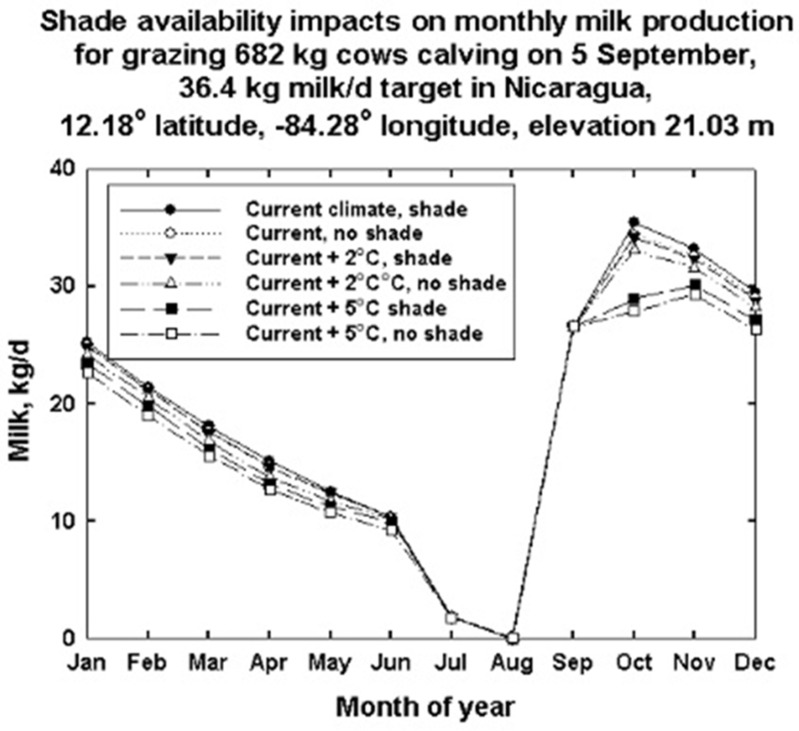
This figure illustrates the impact of shade availability and different assumptions about climate warming of 2 °C and 5 °C. The average day of each month for this location in Nicaragua shows a typical milk production capacity given a target of 36.4 kg of milk per day. The calculations performed for this elevation use global monthly climate data from Table S2 for these geographic coordinates. DNM assumes that these average monthly climate data (air temperature, wind speed, relative humidity and cloud cover) are the same for each day of the month. The hourly values of all the environmental variables are interpolated as a sinusoidal variation of the temperature minimum at sunrise and maximum approximately one hour after solar noon (deviation of times of extremes set by user relative to daily solar maximum and minimum times computed from the embedded solar radiation subroutine, Solrad, in DNM) from maximum/minimum values in Table S2, as described in the description of the microclimate model above.

The process whereby DNM proceeds from an individual day’s simulation to an annual integrated result is illustrated in Figure 9. The average day of a given month is used as a basis for these calculations. Each dot or symbol in Figure 9 represents an integrated 24 h day of climate variation and cow milk production. This is also driven by the lactation stage of a cow’s annual reproductive cycle. The dry down in milk production that anticipates a 5 September calving date dominates the figure. The numerical integration that occurs from the average day of one month to that of the next assumes that all the days in a month are the same in that month. These simulations are for the warmest, most southerly location of the three simulations performed for North and Central America in Nicaragua at 12.18° north latitude, −84.28° West longitude at an elevation of 21 m. Shade versus no shade simulations for current climate, a 2° warming and a 5° climate warming are plotted. Clearly, the stage in the lactation cycle dominates monthly climate and shade availability in terms of daily milk production. The target milk production for the simulation was 36.4 kg of milk per day. Notice that for each climate scenario in terms of temperature, the absence of shade results in lower estimates of milk production.

**Figure 10:**
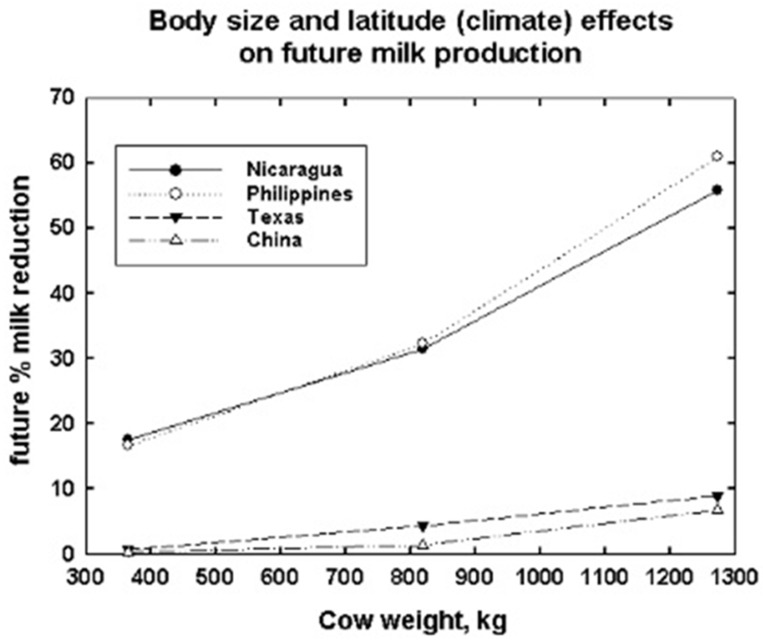
This figure shows the influence of body size and latitude (12° and 30° north latitude) in the Americas and Asia and percent reduction in milk at 12° and 30° north latitude and approximately 30 m elevation.

The percent reduction in future milk production for the two most southerly latitudes in the Americas and the Asian continent are shown in Figure 10. Body size effects on the percent reduction are much greater in the two southerly locations at 12° latitude. It is here that body size differences have the greatest impact on milk production potential. These simulations all assume that the animals are grazing outdoors and have up to 100% shade available to them if they choose to use it.

**Figure 11:**
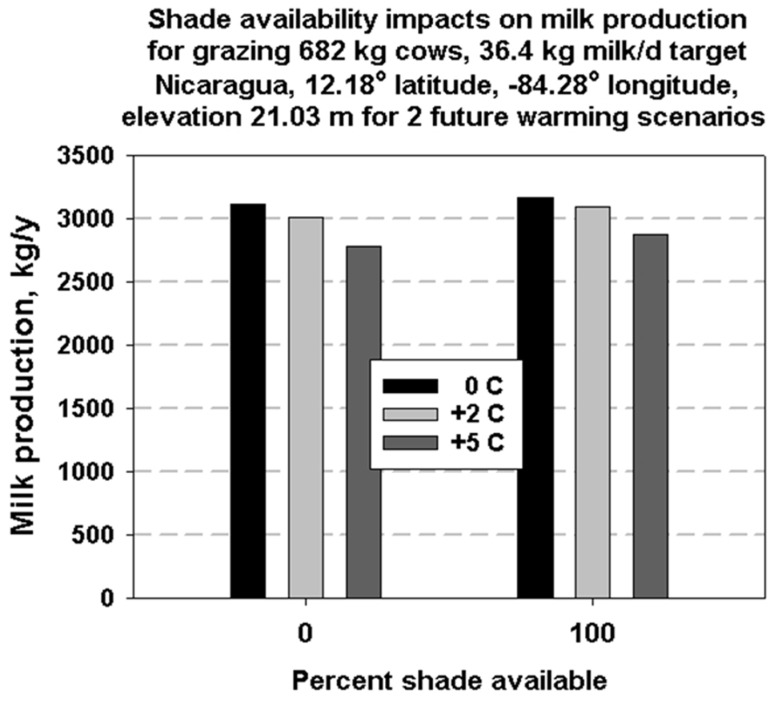
This figure shows shade availability impacts on milk production at a site in Nicaragua integrated over a year for 3 climate scenarios: current, 2 °C warming and a 5 °C warming scenario. The left bar graph in each group shows the effect of shade availability under current and possible 2 and 5 °C warming climate scenarios. The results suggest that climate warming will be nonlinear downward in its suppression of milk production as temperatures rise.

The percent milk production reductions for Nicaragua are shown in Figure 11. This figure shows the effect of both shade availability and increases in temperature on annual milk production potential for the site for 682 kg cows that are 50% black and 50% white.

**Figure 12:**
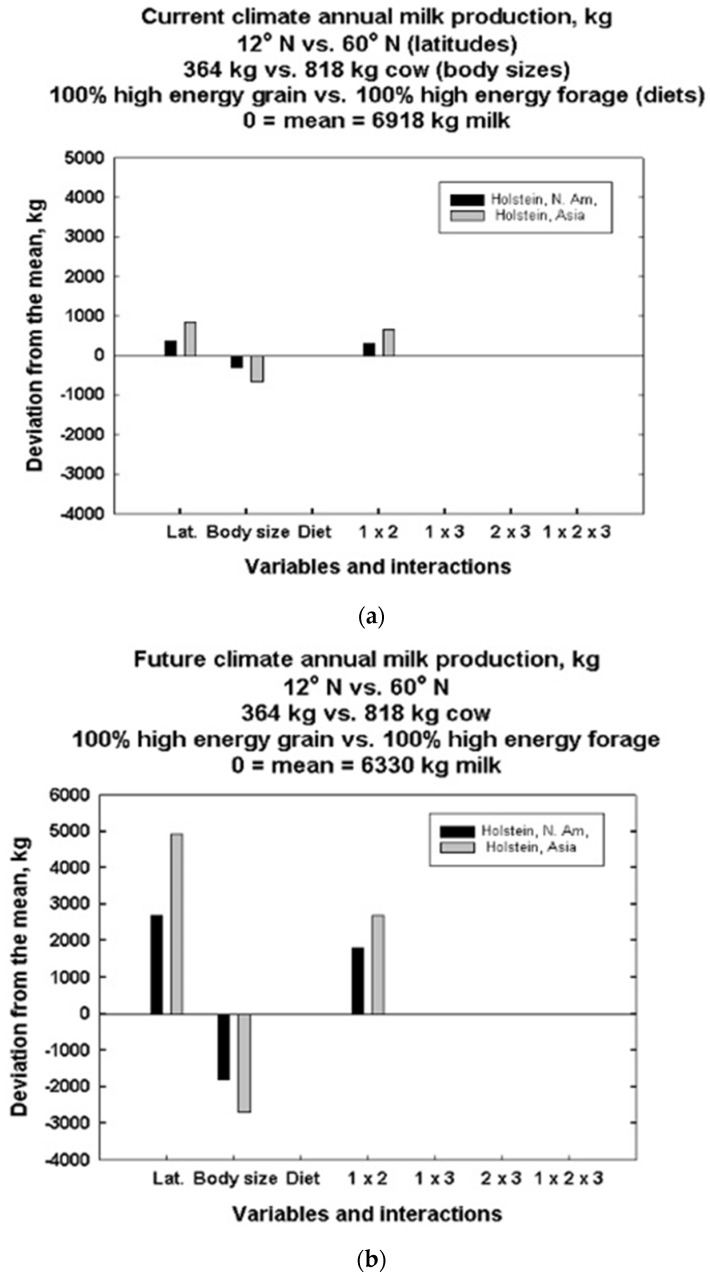
(**a**,**b**). These two graphs reveal main effects and interactions relative to the overall mean of annual milk production in kilograms for simulated sites at 12° north versus 60° north, body sizes of 384 kg versus 818 kg and all high-energy grain diets versus all high-energy forage diets. These Yates algorithm results for milk production changes and water intake changes (Figure 13a,b) are due to differences in latitude, body size and diet calculated by DNM. Notice: (1) the different mean values for each figure, showing overall (experimental design) average, decreases from 6918 to 6330 kg in milk production for a 3 °C warming; (2) milk production becomes much more sensitive to latitude and body size with climate warming. Each vertical bar indicates the positive or negative deviation from the overall mean caused by the variable or interaction effect going from the low to high value. For example, in (**a**), current milk production increases as one proceeds from low to high latitude, whereas it decreases as one proceeds from small to large body size for the same target milk production. In (**b**), climate warming increases sensitivity to the same magnitudes of independent variable changes.

For the global simulations evaluated using the Yates algorithm, the “target” maximum milk production used by DNM was the observed Wisconsin experimental farm average value of 52.4 kg per day that contained 3.12% fat and 2.71% protein. The Yates algorithm analysis of computational results is shown in Figure 12a,b and Table S5. These results illustrate how main effects and interactions of the independent variables for Holsteins are likely to impact milk production and water needs when outdoors with a constant fecal water content of 80% and a 5 September calving date. 

In Figure 12a, the mean milk production for all latitudes, body sizes and diets in the Americas is 6918 kg of milk per year. All effects are plotted relative to the overall mean. Main effects of climate at 12° versus 60° north latitudes are in the two leftmost vertical bars. The leftmost (darker) bar is the Americas, and the next (lighter) bar to the right represents Asia. They are both above the overall mean, but the milk production improvement in Asia is greater than that in the Americas. Both bars show a positive deviation from the mean when comparing 12° versus 60° latitude. The second pair of vertical bars to the right show that increases in body size diminish the overall milk production mean of 6330 kg of milk per year when going from 364 to 818 kg cow sizes. There are no appreciable heat balance effects, but there is an effect on water balance, which affects milk production effects due to diet going from high-energy grain to high-energy forage. There is a positive two-factor interaction effect of the latitude increase and body size increase variables, with the improvement in milk production greater in Asia than in the Americas. The higher-order effects of the first and third, second and third and the three-factor interactions are negligible.

Figure 12b shows that future climate warming scenarios run on the same locations have a mean milk production of 6330 kg of milk per year, down by about 600 kg per year of milk. In current climates, there is an overall mean requirement of 9316 kg of water per year. Nonetheless, the deviations seen in future climate scenarios show much greater deviations, both positive and negative, by factors up to five times prior to variation under current climate scenarios. Once again, there is no diet deviation effect, but the one next to the two-factor interaction, i.e., latitude and body size, is positive as it was before. However, the deviations are greater, and again, Asia has a greater deviation than the Americas.

**Figure 13:**
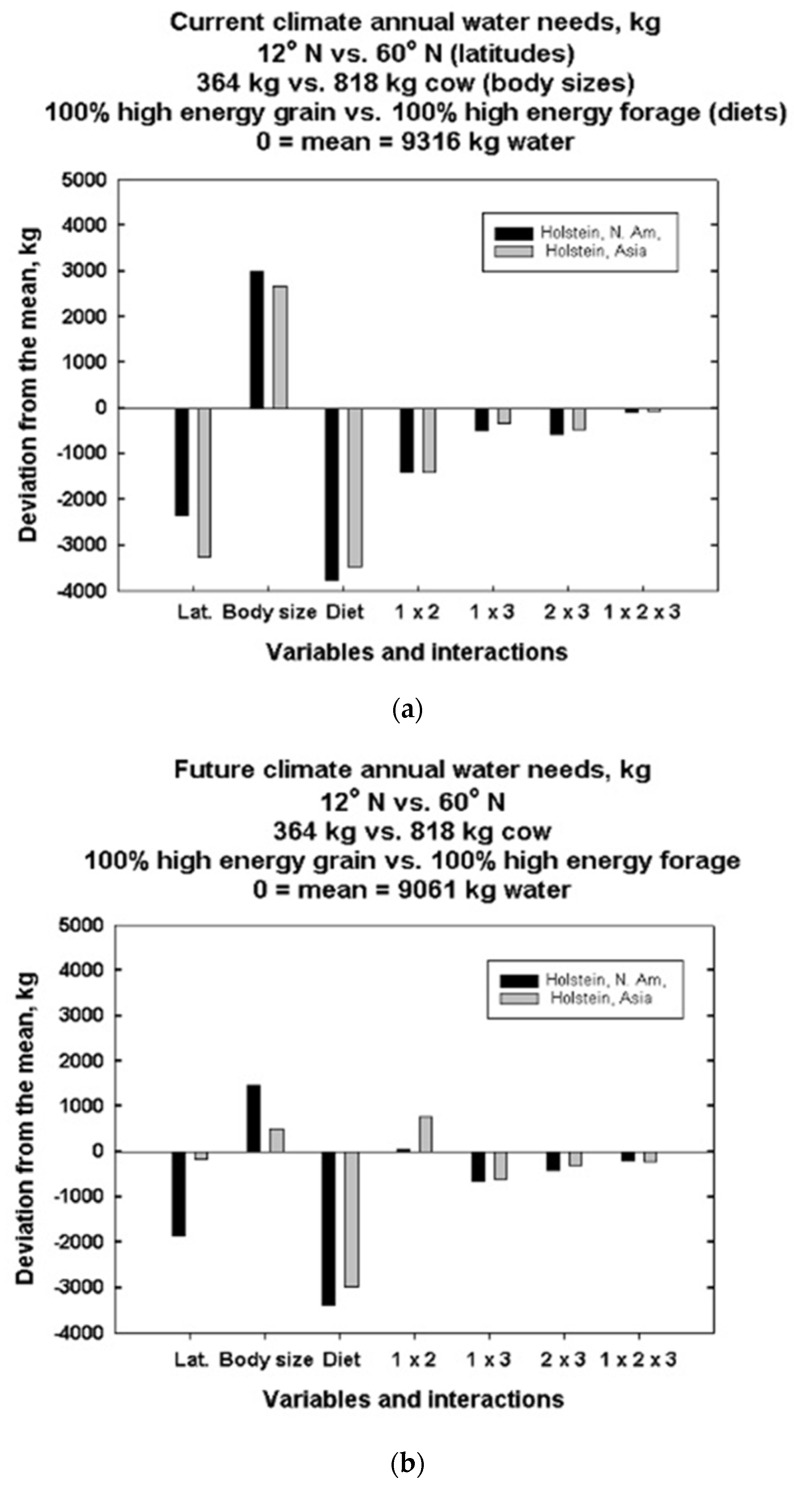
(**a**,**b**). When comparing these two figures against Figure 12a,b, this shows that the average milk/water ratio drops from the current climate at 74% (6918/9316) to 70% (6330/9016) with a 3C climate warming, suggesting more water will be used by the cows for cooling with climate warming.

Each vertical bar indicates the positive or negative deviation from the overall mean caused by the variable or interaction effect going from the low to high value. For example, in Figure 12a, current milk production increases as one proceeds from low to high latitude, whereas it decreases as one proceeds from small to large body size for the same target milk production. In Figure 13b, climate warming increases sensitivity to the same magnitudes of independent variable changes.

Figure 12a and Table S5 show several interesting results about the relative effects of latitude, body size and diet types and their interactions on milk production and water needs for grazing bovines. First, annual current and future milk production in Holsteins is most affected by changes in latitude and body size in the Americas and Asia. Second, we see that annual current water needs of Holsteins are most affected by changes in diet at a given latitude. Third, changes in diet also have the greatest effect on future water intake of Holsteins. Fourth, the different mean values for each figure show an overall (global) average decrease in milk and water from left to right for 3 °C warming. Fifth, milk production becomes much more sensitive to latitude and body size with climate warming. Sixth, the mean annual (milk produced/water need) ratio drops from the current 74% to 70% with 3 °C climate warming, suggesting more water intake will have to go to evaporative cooling and less to milk production. Table S4 shows the greatest changes for feed needs occur outside the middle latitudes belt.

Figure 13a,b shift the focus from milk production to water requirements. In this case, the direction of the deviations for water requirements are the opposite of those for milk production potential. Also, diet now becomes very important for water requirements. Furthermore, all the two-factor and three-factor interactions are now larger. The overall mean water requirements under future climate scenarios for the simulation sites indicate an average annual water requirement of 9316 kg of water per year per cow. However, the mean water requirement for the climate warming scenarios suggests 9061 kg of water required per year on overall average, a difference of about 150 kg of water per year per cow, an approximately 2.7% difference. This may reflect depressed potential milk production due to heat stress.

**Figure 14:**
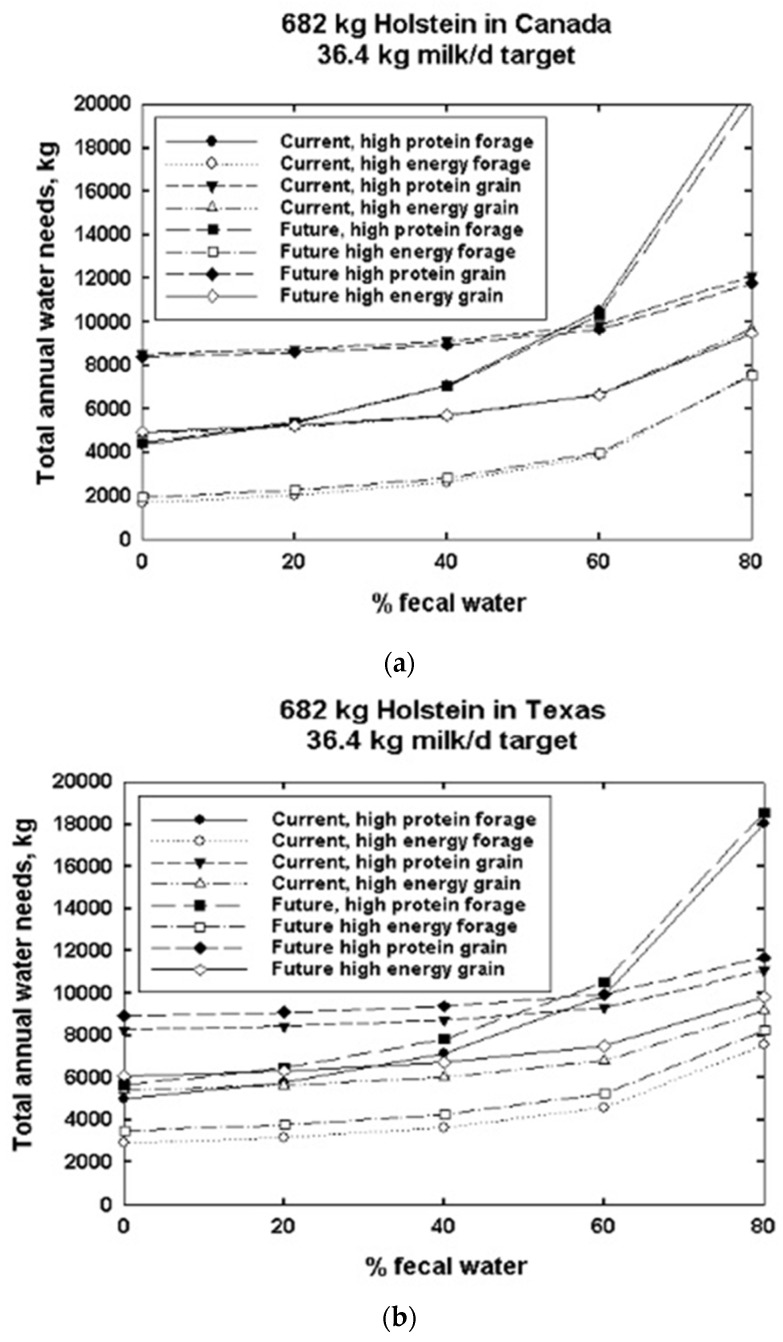

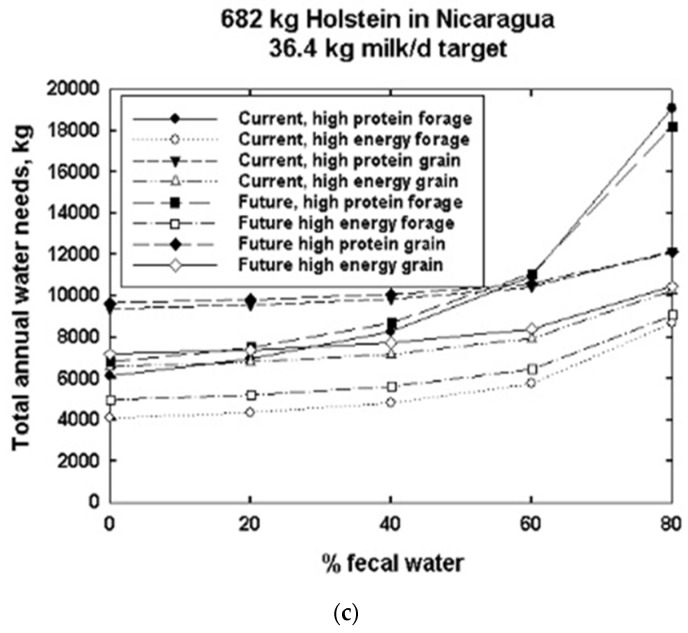
(**a**–**c**). Dairy Niche Mapper-calculated diet and fecal water content effects on water needs for 682 kg Holsteins in North and Central America producing a maximum of 36.4 kg milk/d. Cows were assumed to follow an annual lactation curve (Figure 9) with a maximum production of 36.4 kg/d, e.g., as in (**a**). Canada at ~60 °N latitude; (**b**). USA (Texas) at ~30 °N latitude; (c). Nicaragua at ~12 °N latitude. Note that water intake on the HPF (high-protein forage) diet at 80% fecal water content is constant for current and future times, but the milk production in Table S5 shows a future decrease, suggesting that more water is going to temperature regulation in the future instead of milk production.

Figure 14a–c show how fecal water content interacts with diet water content to affect water needs. The high fecal water content in Holsteins of 80%, plus diet water content, i.e., a high-protein forage (HPF) diet (Table S3), which has less water content (77.5% DM) than a high-energy forage (HEF, 31.5% DM) diet, drives the water needs results for Holsteins outdoors at three latitudes in North America (Figure 14a–c) and the nearly identical plots of the three latitudes in Asia (Figure S8a–c). 

We see that for cows, the 100% high-energy forage diet results in the least annual current and future water needs (Figure 14a–c). 

When cows are at higher latitudes, the differences between the current and future amounts of milk produced are small to absent (Table 3) for a constant calving date of 5 September, which is optimal in the northern hemisphere according to DNM calculations. Even a 5 °C warming at the China site at 30.02 N. latitude shows less than a 1% decline in potential annual milk production. The Russian and Canadian sites at ~60 °N show little impact of climate warming on milk production potential. However, animals at lower latitudes, e.g., the Philippines and Nicaragua at ~12° north latitude, are impacted by a 3 °C climate warming more than animals at higher latitudes. Even a 3 °C warming at higher latitudes greater than 30° will have a small impact on milk production depending on body size, calving time, diet and availability of shade. Future amounts of cow milk production are reduced by up to 60% at the low elevation of the 12 °N latitude sites in Nicaragua and the Philippines (Figure 4, Table 3). The reductions are greatest for the largest body sizes.

## 4. Discussion

It is now possible to “design” animals in silico in three dimensions, analyze the 3D models using CFD to obtain heat and mass transfer coefficients and then insert them into NM or DNM to determine the simultaneous impacts of climate, morphology, physiology and behavior on food and water requirements and milk production potential. Milk production in dairy animals depends on complex, non-linear interactions between correlated and independent variables that include location (latitude, elevation), climate (confinement vs. grazing), animal parameters (morphology, physiology, behavior), month of calving and diet.

Local environments set the boundary conditions for heat and mass exchange between an animal and its environment. Those exchanges enable the maintenance of a constant internal environment in animals that permits the optimization of biochemical and physiological processes to ensure survival, growth and reproduction potential. Milk production, growth potential and immune function depend on having mass and energy resources that exceed those essential for maintenance. Dairy Niche Mapper quantifies the heat and mass balance of dairy animals to allow for a rapid assessment of the feasible bounds of dairy animal functions, given the environmental and bovine characteristics that vary in space and time.

Using “average cows” of different sizes in the simulations allows for an evaluation of where environmental conditions may or may not impinge on efforts to increase and improve the quality of milk production in different areas of the earth.

Historically, there have been a variety of methods of approximating environmental constraints, such as the temperature-humidity heat index, THI [50,51]. Dairy Niche Mapper extends the applicable variable list by adding management options as well as environmental and animal variables, such as elevation and latitude, wind speed, solar and thermal radiation, that impact production potential. Dairy Niche Mapper incorporates animal properties, such as size and shape, which affect surface areas for each of the heat transfer mechanisms. Thus, DNM is not limited to a specific location or size or breed of cow. Each functional heat transfer area changes with postural changes of the cow, hence the importance of accurate morphology descriptions (Figure S2). Haircoat properties, e.g., haircoat depth, hair length, hair density and hair diameter all affect parallel processes of conduction and thermal radiation in haircoat [26,52]. Haircoat color, which is relevant for solar radiation absorption [53], is also included. Because the heat and mass balances are coupled [42], the two balances can be used to compute requisite feed and water needs and potential for milk production as a function of climate, animal and diet properties for different target maximum milk production rates.

The computed outputs of feed and water needs and milk production from DNM are consistent with the limited validation data available for groups of cows (Figure 2 and Figure 3). It is clear from Figure 1 that individual data from cows show variation within body size and level of feed intake in milk production. That variation could be due to health status, response to diet type, endocrine status or other variables. Nonetheless, it provides quantitative insight into average performance in milk production in the context of local climates. For simplicity of calculations and comparisons, day averages of available experimental cow data were used.

Current global warming data are at the higher climate projection estimates, which raises concerns about water needs vs. water availability for dairy animals in the future. Annual future water intake by Holsteins is affected by differences in diet. If dietary and water needs are not met, this will impact milk yield. Variation is seen between Holsteins in North America and Asia when observing the effects of size and latitude (Figure 13 and Figure 14a–c, Table S5) on production potential.

It is interesting to note that the middle latitudes have the least feed needs (Table S4). At 80% fecal water, the middle latitudes have the lowest maximum water needs. The cause seems to be different at low latitudes vs. high latitudes. The highest values are the northerly latitudes where metabolic rate is highest for maintenance. High metabolic rates drive increased respiratory evaporative water loss. At the lowest latitudes, very high temperatures and humidity drive heat stress, higher metabolic rates and, again, high annual water loss values.

### 4.1. General Summary

The value of the DNM is not just in the heat balance and the feed/water requirements that do not all just scale proportionally to the heat balance calculations, as demonstrated by the water requirement figures in this study. Its greatest value is in its generality for broadly understanding how different environments and animal geometries, physiologies and behaviors provide vehicles for affecting survival, growth and reproduction. This model allows for robust quantitative evaluations of mitigation strategies that could provide effective responses to the threats of climate change.

The independent variables, i.e., latitude, body size and diet, have greater impacts on current and future milk production and water needs than the two- or three-factor interactions, as seen in the full factorial analysis of the three independent variables in Figure 12a,b and Table S5. However, the effects, or lack of effects, of certain combinations should be noted. For example, in Table S5, diet type does not affect current or future milk production but has a substantial effect on current and future water needs. This is because milk production calculations using DNM are based on how much milk a cow can produce and still dissipate enough heat to maintain its body temperature. Thus, diet type has no direct influence in estimating milk production, except indirectly, by affecting water balance.

Most of the interaction effects are absent for milk production, but all are important for water needs now and in the future. The three-factor interaction is absent for milk production and is the smallest for water needs of all interaction effects.

Smaller cows are more tolerant of climate changes than larger Holsteins, because they have better convective cooling due to their greater surface-to-volume ratio and their thinner boundary layer. They have a lower water requirement because of less milk volume produced and can tolerate better high humidity and temperature by behavioral and physiological adjustments.

Increasing the latitude from 12° to 30° lowers temperatures and humidity, thereby diminishing the differences between current and future milk production projections.

Increasing elevation reduces air temperatures by 5.5–9 °C/kilometer increase in elevation, making zoning of land use in tropical and subtropical environments of high importance. Our results support the idea that future outdoor managed milk-producing species would more effectively produce milk at higher latitudes or at higher altitudes, especially in tropical and subtropical regions. The greatest differences between current and future input needs and output production are in the low-latitude, low-elevation regions. This is remediated in part by frequent crossbreeding using *Bos indicus* and *Bos taurus*, which results in animals better suited for milk production and with better disease resistance in the low tropics. When temperatures and relative humidity are lower, high-quality forage is more readily available, more water is available, and more hours of foraging activity are feasible.

Simulations with or without shade availability for grazing dairy bovines show the beneficial effects of shade on milk production, especially in the first few months after calving. The absence of shade during these times can result in reduced milk production. Shade, natural or man-made, is a factor in minimizing heat stress by substantially reducing solar radiation load on dairy animals, thereby reducing thermoregulatory water needs and increasing milk production [54].

### 4.2. Some Limitations and Caveats

A live cow operates differently than a DNM cow. The DNM cow maximizes milk production to the greatest extent possible. However, live cows reduce milk production as soon as they are heat-stressed. Thus, our results overestimate actual production in heat stress-inducing conditions. The Dairy Niche Mapper could also be used to identify conditions that induce heat stress at a more refined level than the THI approach (see Supplementary Materials), because it can include feedback and behaviors that can alter local environments that mitigate heat stress.

These results should be viewed as hypotheses to be tested. Despite our extremely limited data in a cold and a warm environment for milk production and the broad range of locations and species testing of Niche Mapper, the basis for DNM, there is still much to be done experimentally to validate in greater detail our current estimates of how climates and animal properties impact reproductive efforts.

The simulations conducted in this study are for steady-state heat and mass transfer. The Dairy Niche Mapper has the capacity to also include transient conditions for both heat and mass, but this is not included here because of space and time constraints.

## 5. Conclusions

The following specific conclusions can be drawn from this study:

1. DNM can define the climate spaces and climate cliffs where target milk production levels are or are not feasible. The climate cliff precipices and their slopes show where and how fast heat stress prevents achieving milk production and even survival. DNM provides an improved quantitative understanding of the multidimensional nonlinear interactions of climate variation and dairy bovine characteristics for current and future milk production as well as feed and water needs for grazing and confinement dairy operations. DNM outputs include feasible activity times, milk production and water and feed needs for different-sized Holstein cows on high-grain (confinement feeding) versus high-forage (grazing feeding) diets at three arbitrary north latitudes, 12°, 30° and 60°, for North and Central America and Asia.
2. Trade-offs in climate, location, body size and diet type as main and interactive effects on cow feed and water needs and the capacity to produce animal products for human consumption are illustrated. Relocation efforts and selection or crossbreeding for smaller animal size are needed for grazing dairy animals, especially in low-elevation tropical and subtropical regions to maintain or increase production capabilities as climates continue to warm.
3. Selection of smaller sized and high production efficiency cows may be more suitable for tropical and subtropical regions, because these cows can ameliorate some of the effects of climate warming at low elevations.
4. Temperature, body size and, to a lesser extent, wind speed, relative humidity, solar radiation and haircoat color affect the ability to produce milk at targeted levels for cows. It is clear that it is possible, by adjusting cow morphological and physiological properties and behavioral traits, in combination with modifications of land use and management practices, e.g., season of calving and utilizing bioinformatics-biophysical computational models, to rapidly advance our understanding of better options for maintaining and improving dairy production globally in the face of the current escalating global climate crisis.

## Figures and Tables

**Table 1:** Sample Dairy Niche Mapper inputs for a Holstein cow.

	Input	References
Animal Attributes		
Height (cm)	147.3	Our measurements
Fur depth (cm)	0.336	Our measurements
Hair length (cm)	0.635	Our measurements
Weight (kg)	682	Assumed value
Calving month	9	Assumed value
Calving day	5	Assumed value
Shade available	Yes	Assumed value
Fur reflectivity %, % of total skin		
Black	16, 50	Our measurement, assumed % portion
White	48, 50	Our measurement, assumed % portion
Brown or Red	40, 0	Our measurement, assumed % portion
Tan	45, 0	Our measurement, assumed % portion
% Fecal water	80.	Our measurements
Location and Elevation		
Latitude (deg)	12.03	niche-mapper.com/Global Climate Extractor
Longitude (deg)	123.77	niche-mapper.com/Global Climate Extractor
Elevation (m)	27.72	niche-mapper.com/Global Climate Extractor
Milk Production		
Maximum Expected Milk (kg)	36.4	From typical Holstein production range
% Milk fat	3.2	From typical Holstein production range
% Milk protein	3.5	From typical Holstein production range
Barn Usage	No	
Summer Diet		
High-Protein Forage (HPF)	100	Assumed diet % value
High-Energy Forage (HEF)	0	Assumed diet % value
High-Protein Grain (HPG)	0	Assumed diet % value
High-Energy Grain (HEG)	0	Assumed diet % value
Winter Diet		
High-Protein Forage	100	Assumed diet % value
High-Energy Forage	0	Assumed diet % value
High-Protein Grain	0	Assumed diet % value
High-Energy Grain	0	Assumed diet % value

Summer versus winter diet refers to user choices regarding percent of different diets as defined in Table S3. The 100% high-protein forage diet was selected because it could apply equally well to grazing or confined dairy cows, since it is made up of alfalfa, hay and no corn, although some wheat, barley or oats are present.

**Table 2:** Experimental design defining variable values.

	2^3^ Full Factorial Design Levels with Center Replicate—Holsteins		
Variable	Low (−)	Center (0)	High (+)
Latitude (degrees N)	12° Philippines	30° China	60° Russia
Size (kg)	364	818	1273
Diet (% energy diet type)	100% HEG	50/50 HEG/HEF	100% HEF

HEG = high-energy grain; HEF = high-energy forage (Table S3).

**Table 3:** Holstein annual current and future milk production calculations for body size and location effects using DNM. Annual milk production follows a typical annual milk production cycle tied to a 5 September calving date with a 36.4 kg/d milk maximum target, e.g., as in Figure 9.

	Location					
Milk Production, kg/y (Body Size, kg)	Philippines	China	Russia	Nicaragua	Texas	Canada
Current Milk (364 kg)	6864	7008	7008	6897	6967	7008
Future Milk (364 kg)	5669	6988	7008	5753	6921	7008
Current Milk (818 kg)	6576	7008	7008	6630	6967	7008
Future Milk (818 kg)	4506	6915	7008	4494	6670	7008
Current Milk (1273 kg)	5659	6970	7008	5729	6886	7008
Future Milk (1273 kg)	2496	6503	7008	2236	6272	7008

## Data Availability

All data used in the calculations are in the tables and figures of this report. Detailed descriptions of the endotherm equations may be found in [26,28]. A generic version of the microclimate and endotherm animal models may be found at NicheMapR (https://mrke.github.io/, accessed on 14 May 2023). DNM is currently available from the corresponding author.

## Supplementary Materials

The following supporting information can be downloaded at: https://www.mdpi.com/article/10.3390/ani13193043/s1, Table S1: Global Simulation Locations; Table S2: Average monthly climate data for 6 simulation locations; Table S3: Cow diet categories and properties (NRC Table 15-1); Table S4: Dairy Niche Mapper calculations for a 682 kg Holstein annual diet needs—current and future; Table S5: Yates algorithm analysis of DNM results for latitude, body size and diet effects; Figure S1: Simulation locations for North America and Asia for the Yates’ algorithm analyses of how latitude, bovine body size and diet type can affect feed and water needs and milk production potential in outdoor environments; Figure S2: (a) Overview of the heat energy balance on a cow outdoors, (b–d) The side, front and top virtual cow views; Figure S3: 3D representation of the wet weight food needed to maintain a 50 kg per day milk production target by a 643 kg black Holstein cow; Figures S4 and S5: Water needs for a black (top) versus white (bottom) Holstein in the same environment; Figure S6: (a–d) Water loss for 643 kg, 100% white Holsteins for variation in humidity (L/R columns) and solar radiation (Top/Bottom rows), (e,f) demonstrate how haircoat color can modify water requirements as wind and temperatures vary; Figure S7: Effect of relative humidity and coat color on milk production potential of a 643 kg black (top) (a,b) vs. white Holstein (bottom) (c,d); Figure S8: (a,b) “THI effects” of solar radiation and wind speed, (c) looks the same at 2.9 m/s, but at 2.8 m/s, (d) there is a catastrophic drop in milk production potential that allows for less than 10% relative humidity for any milk production at all. (e) shows that the ‘plain’ of milk production is achievable if the solar radiation is decreased to 480 W/m^2^ at 2.8 m/s wind; Figure S9: (a–c) Latitude effects on total annual water needs as affected by percent fecal water and diet type in current climate and future 3° C warmer climates.
