# Supplementary material for: Climate Spaces and Cliffs: A Novel Bovine Thermodynamic and Mass Balances Model
Source: Animals (Basel). 2023 Sep 27;13(19):3043. doi: 10.3390/ani13193043 (PMC10572002; doi:10.3390/ani13193043)
Supplement: Supplementary file 1 [file animals-13-03043-s001.zip › animals-2426347-supplementary.pdf]

Supplementary Materials—Tables

Table S1. Global Simulation Locations

Location	Coordinates and Elevation		
	Latitude	Longitude	Elevation (m)
Philippines	12.03	123.77	27.72
China	30.02	114.32	27
Russia	60.14	166.33	22.56
Nicaragua	12.18	-84.28	21.03
Texas, USA	30.12	-94.68	23.52
Canada	60.11	-95.04	36

Tables S2. Average monthly climate data for 6 simulation locations

Month	Max. Temp °C	Min. Temp °C	Max. Rel. Humid %	Min. Rel. Humid %	Wind Speed (10 m) m/s	Cloud Cover %	Rainfall mm/mo
Jan	29.4	21.0	81.2	60.2	2.6	45.6	55.5
Feb	30.0	21.0	80.7	57.7	2.5	39.5	26.6
Mar	31.4	22.0	78.2	55.5	2.4	33.8	30.8
Apr	32.3	22.9	100.0	53.4	2.4	30.7	61.5
May	32.2	23.0	100.0	56.9	2.0	41.5	167.9
Jun	30.6	22.4	100.0	62.3	2.1	52.2	266.4
Jul	29.9	22.1	100.0	65.5	2.2	57.5	324.4
Aug	29.7	22.1	100.0	66.1	2.3	58.8	344.1
Sep	30.1	22.1	100.0	65.1	1.9	54.1	286.7
Oct	30.1	22.1	100.0	64.1	2.2	47.9	300.0
Nov	30.2	22.0	100.0	62.0	2.3	45.8	191.8
Dec	29.6	21.4	100.0	60.4	2.6	49.1	119.9
AVE	30.5	22.0	95.0	60.8	2.3	46.4	181.3

Table S2a. Philippine site climate. Data for all sites from (New et al., 2002)

Month	Max. Temp °C	Min. Temp °C	Max. Rel. Humid %	Min. Rel. Humid %	Wind Speed (10m) m/s	Cloud Cover %	Rainfall mm/mo
Jan	8.3	-0.5	100.0	55.5	2.5	61.7	35.4
Feb	9.8	1.4	100.0	57.6	2.7	65.4	58.4
Mar	14.8	6.1	100.0	59.4	2.7	67.0	95.1
Apr	21.1	12.2	100.0	58.2	2.6	62.5	140.6
May	26.4	17.6	100.0	58.1	2.4	58.1	163.1
Jun	29.9	21.8	100.0	60.1	2.3	55.0	210.1
Jul	33.0	25.2	100.0	61.5	2.6	43.3	161.1
Aug	33.0	24.6	100.0	59.7	2.4	38.0	124.7
Sep	28.0	19.4	100.0	58.9	2.5	51.4	84.1
Oct	22.8	13.4	100.0	56.7	2.3	53.9	84.7
Nov	16.5	7.2	100.0	54.6	2.4	55.1	58.2
Dec	10.6	1.4	100.0	51.7	2.4	56.0	29.2
AVE	21.2	12.5	100.0	57.7	2.5	55.6	103.7

Table S2b. China site climate

Month	Max. Temp	Min. Temp	Max. Rel. Humid	Min. Rel. Humid	Wind Speed (10m)	Cloud Cover	Rainfall
	°C	°C	%	%	m/s	%	mm/mo
Jan	-11.1	-18.0	100.0	70.7	6.7	72.2	33.9
Feb	-10.9	-18.0	100.0	70.9	6.8	60.4	26.9
Mar	-9.1	-16.8	100.0	66.4	5.9	49.7	23.2
Apr	-4.0	-11.5	100.0	62.8	4.9	51.2	23.1
May	4.3	-2.1	100.0	62.5	4.0	60.4	26.4
Jun	11.2	4.2	100.0	57.9	3.9	61.0	33.6
Jul	14.4	8.1	100.0	61.9	3.9	65.5	57.6
Aug	14.2	8.1	100.0	62.9	3.8	64.9	64.1
Sep	9.9	3.6	100.0	61.5	3.8	62.5	51.5
Oct	1.2	-4.6	100.0	64.7	4.7	63.2	52.3
Nov	-7.4	-13.7	100.0	67.7	5.9	69.1	47.7
Dec	-10.9	-17.8	100.0	67.7	6.6	81.6	33.5
AVE	0.2	-6.5	100.0	64.8	5.1	63.5	39.5

Table S2c. Russia site climate

Month	Max. Temp	Min. Temp	Max. Rel. Humid	Min. Rel. Humid	Wind Speed (10m)	Cloud Cover	Rainfall
	°C	°C	%	%	m/s	%	mm/mo
Jan	29.0	21.8	82.0	65.4	4.3	51.9	171.1
Feb	30.0	21.8	80.6	61.0	4.1	43.8	94.2
Mar	31.4	22.6	79.9	59.5	4.1	41.0	49.8
Apr	31.9	23.5	99.8	56.5	4.1	41.2	62.5
May	31.1	23.5	100.0	62.7	3.4	43.5	208.7
Jun	29.8	22.6	100.0	65.5	3.2	52.2	445.2
Jul	29.9	23.1	100.0	68.4	3.5	58.4	533.0
Aug	29.6	22.5	100.0	67.0	3.2	57.8	431.1
Sep	29.1	22.0	100.0	68.0	2.7	53.4	287.8
Oct	29.3	21.9	100.0	64.0	2.7	53.9	296.1
Nov	28.7	21.5	100.0	65.5	3.3	51.7	243.0
Dec	28.6	21.4	100.0	63.0	4.0	46.5	211.6
AVE	29.9	22.4	95.2	63.9	3.6	49.6	252.8

Table S2d. Nicaragua site climate

Month	Max. Temp	Min. Temp	Max. Rel. Humid	Min. Rel. Humid	Wind Speed (10m)	Cloud Cover	Rainfall
	°C	°C	%	%	m/s	%	mm/mo
Jan	15.8	5.4	100.0	54.6	4.5	56.1	99.7
Feb	18.1	7.2	100.0	52.6	4.7	48.3	95.1
Mar	22.0	10.6	97.4	51.8	4.9	47.4	84.2
Apr	25.9	15.1	100.0	51.2	4.9	45.4	81.8
May	29.4	18.6	100.0	53.3	4.4	39.2	147.4
Jun	32.5	22.0	100.0	54.0	3.9	32.0	146.0
Jul	33.9	23.1	100.0	53.7	3.4	31.6	117.5
Aug	34.2	22.9	100.0	52.8	3.2	34.1	108.2
Sep	31.5	20.4	100.0	53.4	3.6	36.4	147.5
Oct	27.5	14.5	100.0	48.2	3.7	33.9	106.3
Nov	22.4	10.6	100.0	50.3	4.2	45.0	121.7
Dec	17.9	6.8	100.0	51.5	4.3	52.7	121.8
AVE	25.9	14.8	99.8	52.3	4.1	41.8	114.8

Table S2e. US, Texas site climate

Month	Max. Temp	Min. Temp	Max. Rel. Humid	Min. Rel. Humid	Wind Speed (10m)	Cloud Cover	Rainfall
	°C	°C	%	%	m/s	%	mm/mo
Jan	-24.2	-33.0	100.0	46.5	6.3	66.2	14.4
Feb	-21.5	-31.5	100.0	44.5	6.0	52.7	10.2
Mar	-15.2	-27.4	100.0	44.7	5.7	49.3	15.0
Apr	-5.7	-16.9	100.0	48.1	5.7	49.9	19.6
May	3.5	-6.3	100.0	56.7	5.4	60.2	26.5
Jun	12.2	1.2	100.0	54.8	5.0	56.1	38.7
Jul	18.4	6.8	100.0	52.4	4.8	49.5	50.2
Aug	16.5	6.6	100.0	57.2	4.9	53.0	55.6
Sep	9.8	1.8	100.0	62.6	5.6	73.0	49.6
Oct	0.2	-6.3	100.0	64.4	6.1	80.1	42.5
Nov	-11.4	-19.5	100.0	55.2	6.1	75.6	29.6
Dec	-20.5	-28.6	100.0	48.7	5.7	77.2	16.7
AVE	-3.2	-12.8	100.0	53.0	5.6	61.9	30.7

Table S2f. Canada site climate

Table S3. Cow diet categories and properties (NRC Table 15-1)

Category		% dry matter	% protein	% fat	% carb, NDF + starch
High Protein Forage		% as is	%DM	%DM	%DM
(HPF)	Alfalfa-legumes -hay				
	Alfa hay pre bloom	90.0	20.0	3.8	42.0
	Alfa hay mid bloom	90.0	17.0	2.6	47.8
	Alfa hay full bloom	90.0	15.0	2.0	51.8
	Category Average	90.0	17.3	2.8	47.2
	Young grasses				
	Grass, pasture early	27.0	19.5	4.0	55.5
	Grass, pasture late	45.0	9.0	2.8	70.0
	Grass hay mature	89.0	10.0	2.3	72.0
	Category Average	53.7	12.8	3.0	65.8
	Young cereal crops (wheat, barley, oats)				
	Barley grain	88.0	12.9	2.1	76.9
	Oat grain	89.0	11.7	5.4	74.4
	Wheat grain	89.0	12.0	1.9	78.3
	Category Average	88.7	12.3	3.8	75.7
Overall Average		77.5	14.1	3.0	63.2
High Protein Grains and Byproducts					
(HPG)	Soybean meal 48%	89.0	55.0	1.0	16.0
	Canola seed	90.0	20.5	40.5	17.8
	Meat meal	94.0	60.0	9.7	0.0
	Fish meal	92.0	67.7	10.5	0.0
	Cottonseed meal	91.0	45.6	1.3	28.5
Overall Average		91.2	49.8	12.6	12.5
High Energy-Low Protein Forage (HEF)					
	Corn silage 10% grain	35.0	9.0	3.0	71.0
	Sorghum silage	28.0	10.2	3.0	66.1

Overall Average		31.5	9.6	3.0	68.6
High Energy-Low Protein Grains		% as is	% protein	% fat	% carb, NDF+starch
(HEG)	Corn grain, cracked	88.0	9.3	4.3	79.5
	Milo (grain sorghum)	87.0	9.7	3.4	81.4
	Barley grain	88.0	12.9	2.1	76.9
	Oat grain	89.0	11.7	5.4	74.4
	Wheat grain	89.0	12.0	1.9	78.3
Overall Average		88.0	10.9	3.8	78.1
Medium Protein-Fiber Forage					
(MPF)	Mature cereal crops with grain in milky stage				
	Barley silage, headed	35.5	12.0	3.5	56.3
	Oat hay, headed	91.9	9.1	2.2	58.0
	Wheat hay, headed	86.8	9.4	1.7	61.1
Overall Average		71.4	10.2	2.5	58.5
Medium Protein-Fiber Grains					
(MPG)	Most byproducts	81.7	15.8	8.2	48.7
	Whole cottonseed	92.0	23.0	20.0	45.6
	Wheat middlings	89.0	18.4	4.9	60.3
Overall Average		87.6	19.1	11.0	51.5

Table S4. Dairy Niche Mapper calculations for a 682 kg Holstein annual diet needs – current and future

Feed Type - Location	Annual Feed Totals (kg.)		
	Current	Future	% change
Philippines			
High Protein Forage	4942	3920	20.7
High Energy Forage	4845	3843	20.7
High Protein Grain	2782	2207	20.7
High Energy Grain	3322	2635	20.7
Nicaragua			
High Protein Forage	5066	4453	12.1
High Energy Forage	4967	4366	12.1
High Protein Grain	2852	2507	12.1
High Energy Grain	3406	2994	12.1
China			
High Protein Forage	5127	5062	1.3
High Energy Forage	5027	4963	1.3
High Protein Grain	2886	2850	1.3
High Energy Grain	3446	3403	1.3
Texas			
High Protein Forage	5119	5035	1.6
High Energy Forage	5019	4937	1.6
High Protein Grain	2882	2835	1.6
High Energy Grain	3453	3385	2.0

Russia			
High Protein Forage	5837	5535	5.2
High Energy Forage	5723	5427	5.2
High Protein Grain	3286	3116	5.2
High Energy Grain	3924	3721	5.2
Canada			
High Protein Forage	6521	6154	5.6
High Energy Forage	6393	6034	5.6
High Protein Grain	3671	3465	5.6
High Energy Grain	4383	4137	5.6

Table S5. Yates algorithm analysis of DNM results for latitude, body size and diet effects. Interactions, e.g., 1x2 is latitude x body size interaction. 5 September calving.

	Holsteins:: N. America., kg	Holsteins: Asia, kg
**Effects on Current Annual Milk**		
1) Latitude	361.	850.
2) Body size	-317.	-662.
3) Diet	0.	0.
1x2	317.	662.
1x3	0.	0.
2x3	0.	0.
1x2x3	0.	0.
**Effects on Future Annual Milk**	Holsteins: N. America., kg	Holsteins: Asia, kg
1) Latitude	2712.	4944.
2) Body size	-1798.	-2692.
3) Diet	0	0
1x2	1798.	2692.
1x3	0	0
2x3	0	0
1x2x3	0	0
**Effects on Current Annual Water**	Holsteins:: N. America., kg	Holsteins: Asia, kg
1) Latitude	-2334.	-3240.
2) Body size	2989.	2658.
3) Diet	-3754.	-3457.
1x2	-1407.	-1405.
1x3	-497.	-326.
2x3	-566.	-461.
1x2x3	-78.	-48.
**Effects on Future Annual Water**	Holsteins:: N. America., kg	Holsteins: Asia, kg
1) Latitude	-1846.	-178.
2) Body size	1462.	505.
3) Diet	-3377.	-2995.
1x2	53.	763.
1x3	-640.	-605.
2x3	-403.	-292.
1x2x3	-206.	-214.

Supplementary Material - Figures

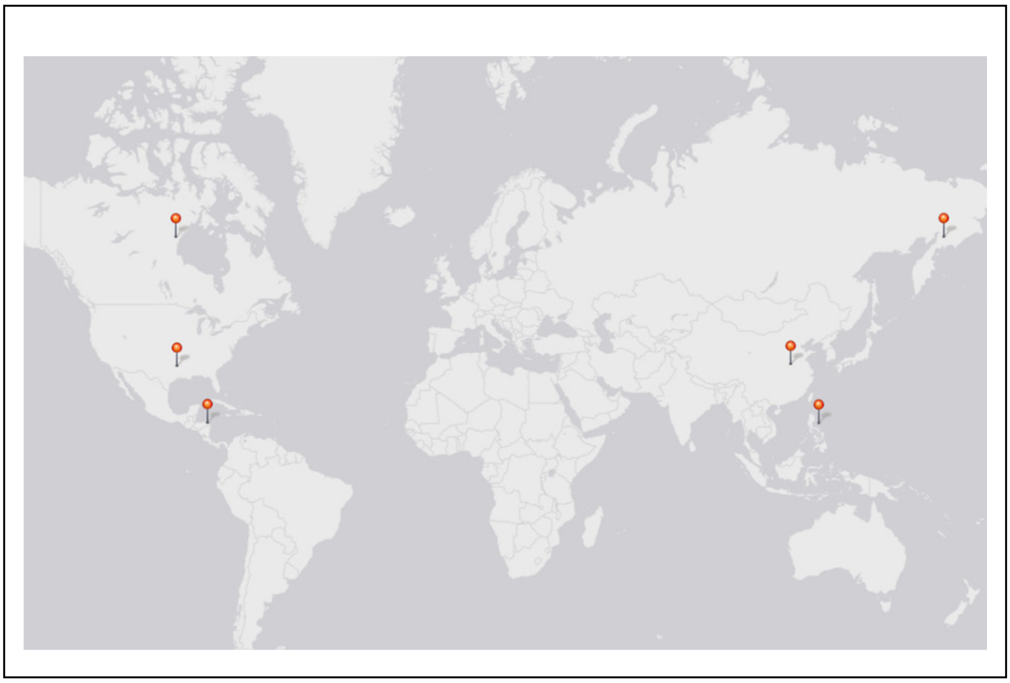

Figure S1. Simulation locations for North America and Asia for the Yates' algorithm analyses of how latitude, bovine body size and diet type can affect feed and water needs and milk production potential in outdoor environments. Table S1 shows the geographic coordinates and elevations.

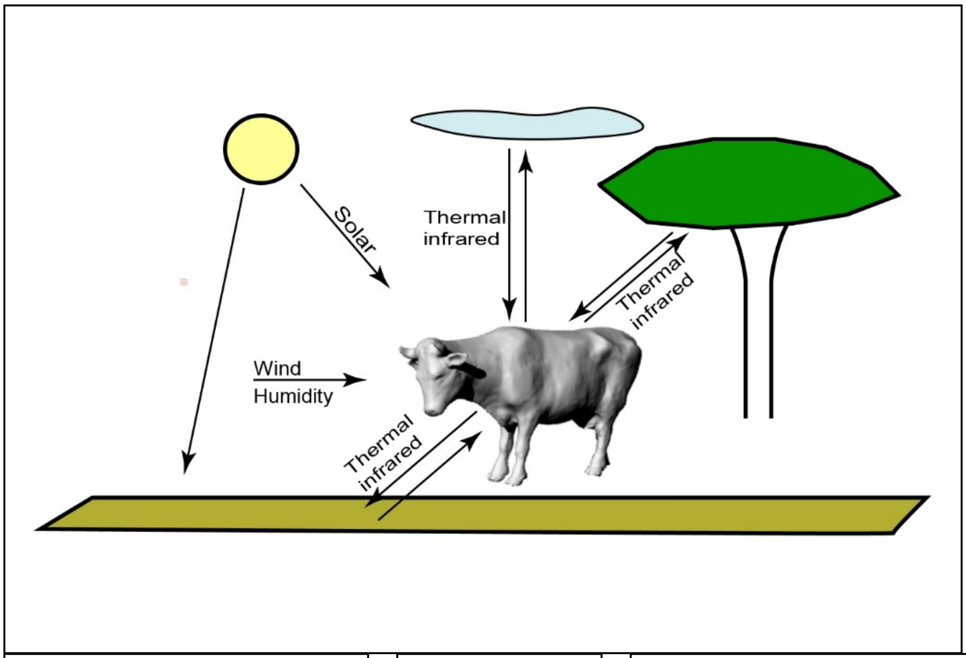

Figure S2a

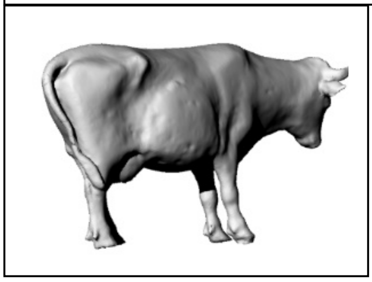

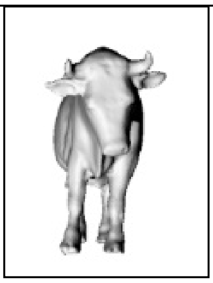

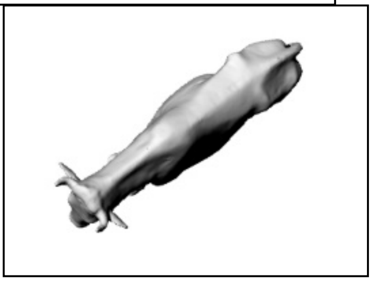

Figure S2b Figure S2c Figure S2d

Figure S2a. Overview of the heat energy balance on a cow outdoors. The top cow image is an oblique view of the 3D image used to obtain 1) the heat and mass transfer coefficients via ANSYS Fluent as described in Dudley et al.(Dudley et al., 2013), 2) the surface, silhouette areas and volume of the cow for any height and weight. The other three figures, S2b, S2c and S2d, are the side, front and top virtual cow views. Dairy Niche Mapper allows for isometric and non-isometric scaling via user inputs of weight and height of a cow. The program automatically checks for consistency of mass, density and volume and corrects the anatomy isometrically or radially depending on user specifications.

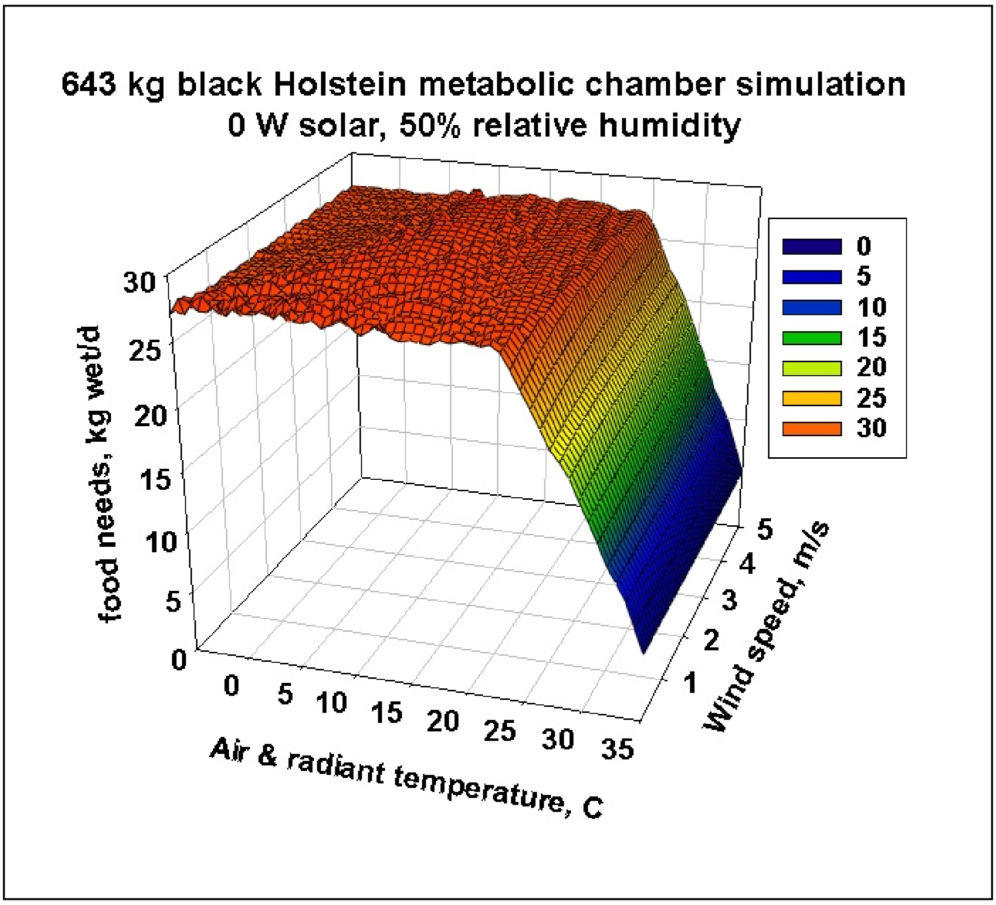

Figure S3. 3D representation of the wet weight food needed to maintain a 50 kg per day milk production target by a 643 kg black Holstein cow. Food consumption is projected to begin to decline at approximately 25 °C. At the lowest windspeeds near 0.1 m/s the transition to reduced feed intake begins at slightly lower temperatures than at higher wind speeds and temperatures as indicated by the horizontal lines on the declining food needs surface.

Figure S4

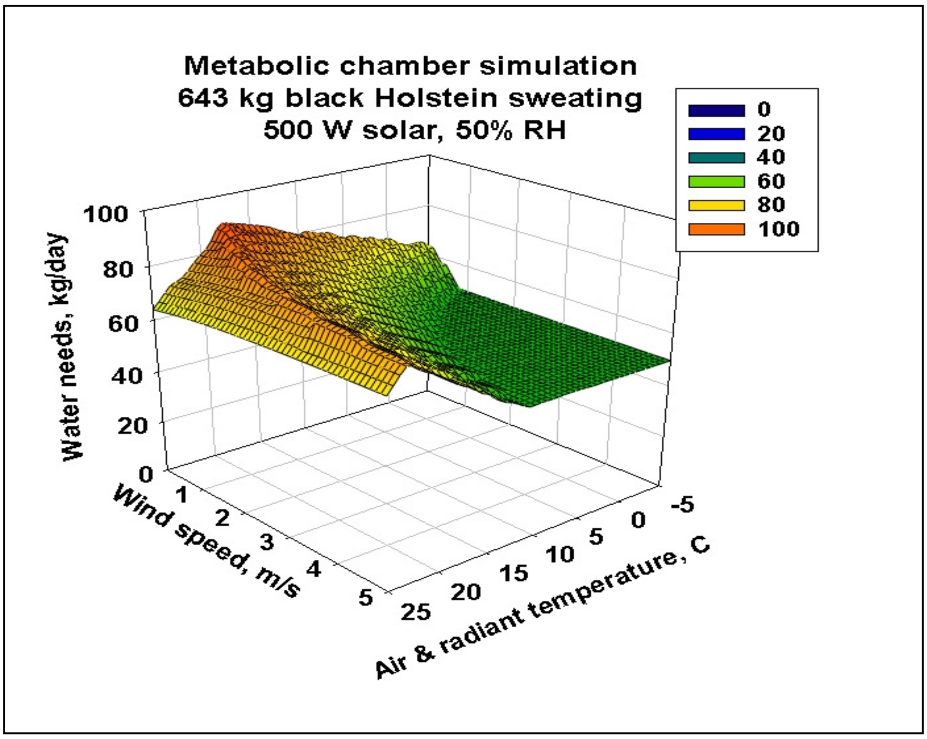

Figure S5

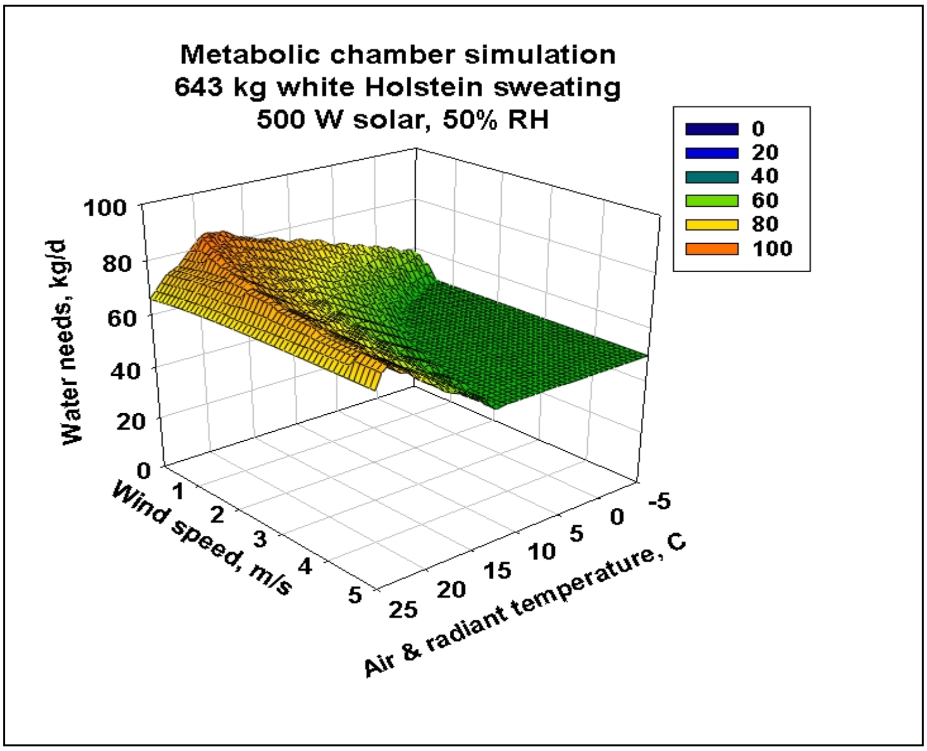

Water needs for a black (top) versus white (bottom) Holstein in the same environment.

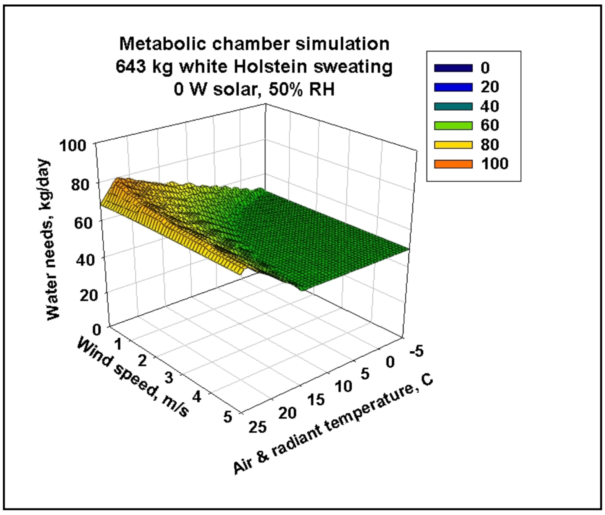

Figure S6a

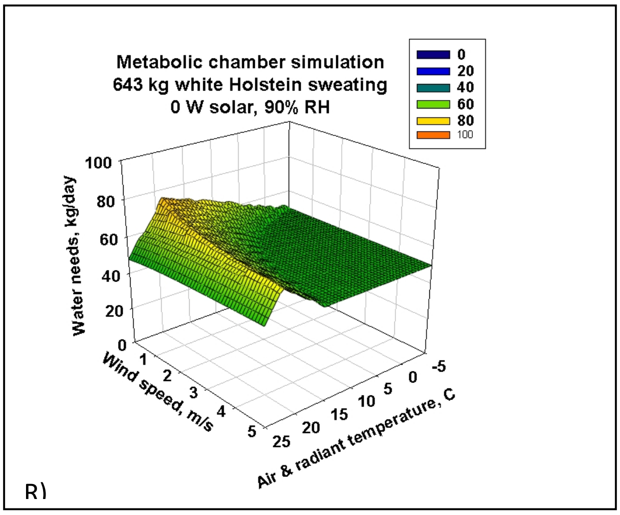

Figure S6b

Water loss for 643 kg, 100% white Holsteins for variation in humidity (L/R columns) and solar radiation (Top/Bottom rows)

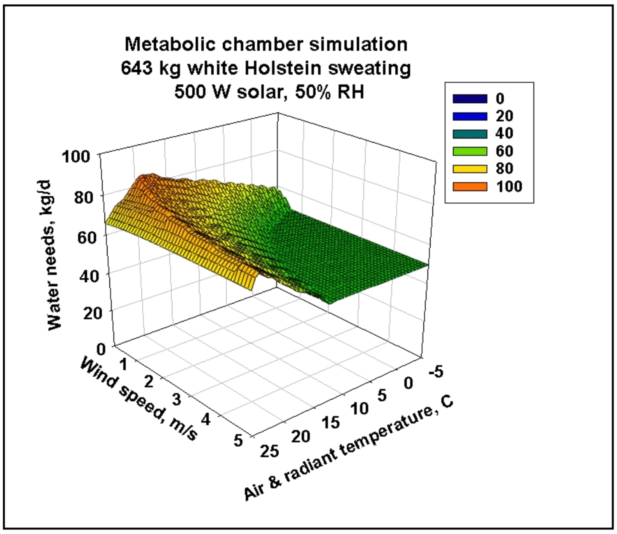

Figure S6c

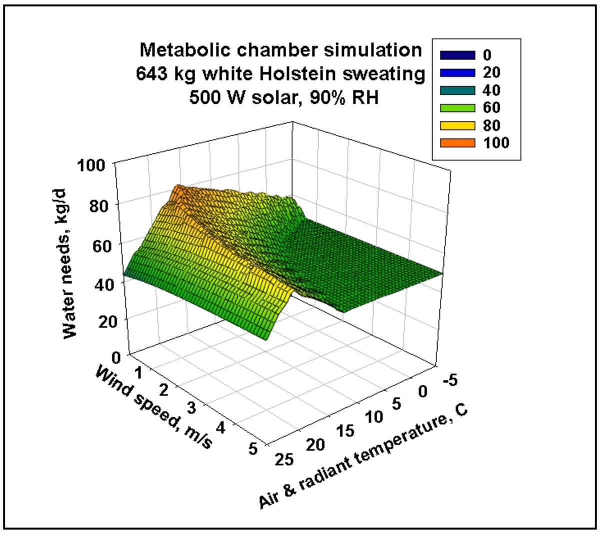

Figure S6d

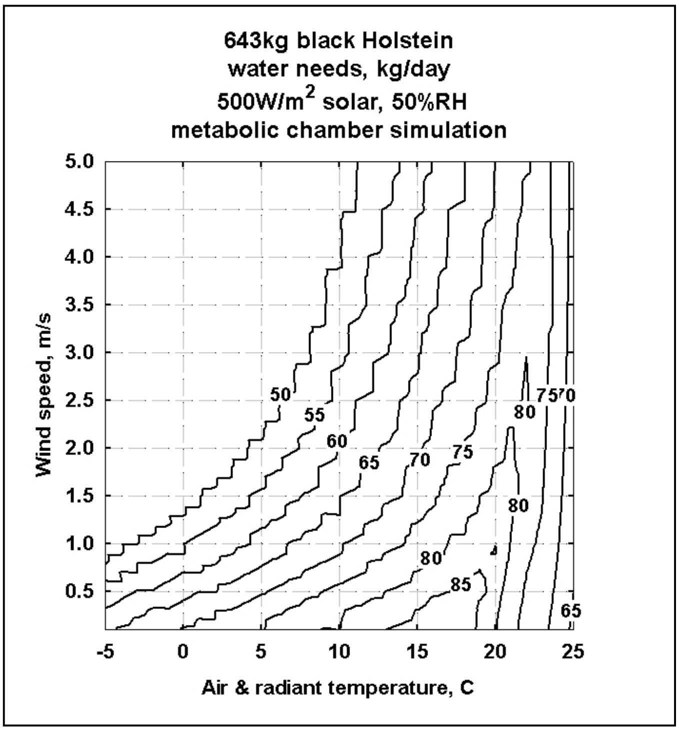

Figure S6e

Contour plots allow for explicit evaluations of responses to multiple variables affecting the response. Figures S6e and S6f demonstrate how haircoat color can modify water requirements as wind and temperatures vary. They also show where climate cliffs occur on the right side of the 'ridge' on these plots where the ridge peak runs from ~17 °C at 0.1 m/s to ~24 °C at 5 m/s. The flat 'plain' of 50 kg/d water loss in the upper left portion of the graph is slightly larger for the white than the black Holstein.

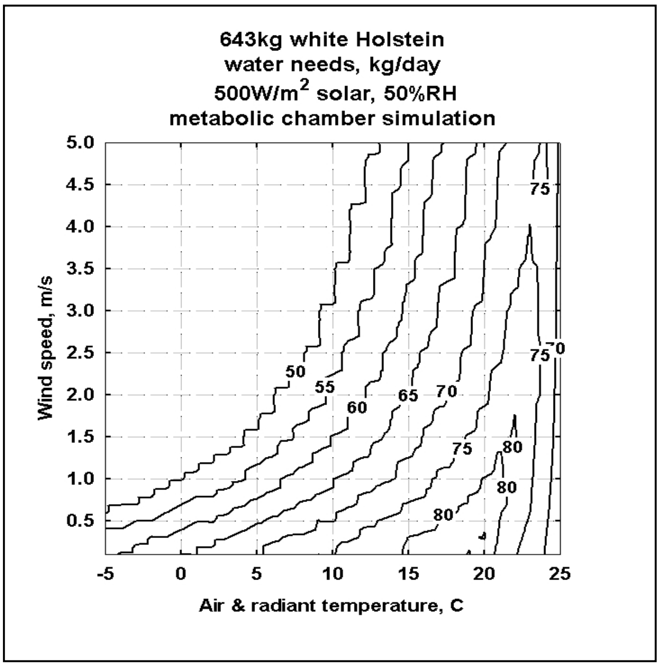

Figure S6f

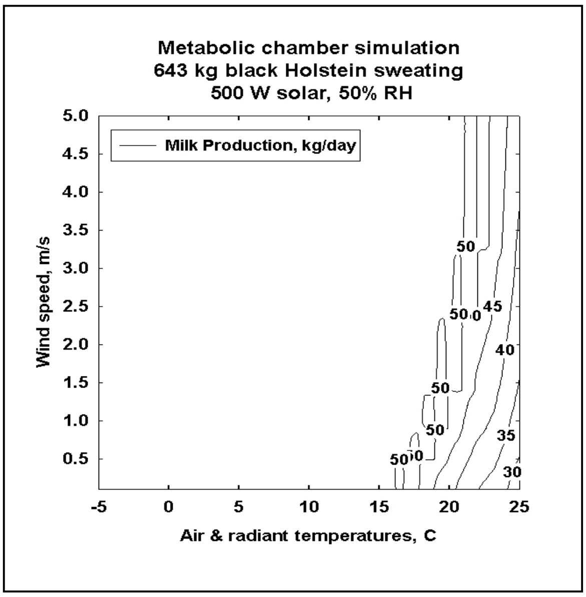

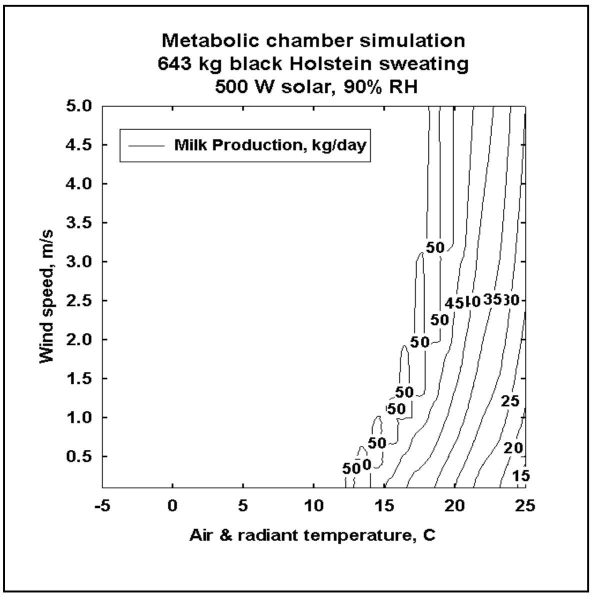

Figure S7a Figure S7b

Effect of relative humidity and coat color on milk production potential of a 643 kg black (top) vs. white Holstein (bottom). The large white area on the left part of the contour plots is the "plain" of where the target 50 kg milk production per day can occur. Contour lines on the right indicate the "cliff" where function is failing.

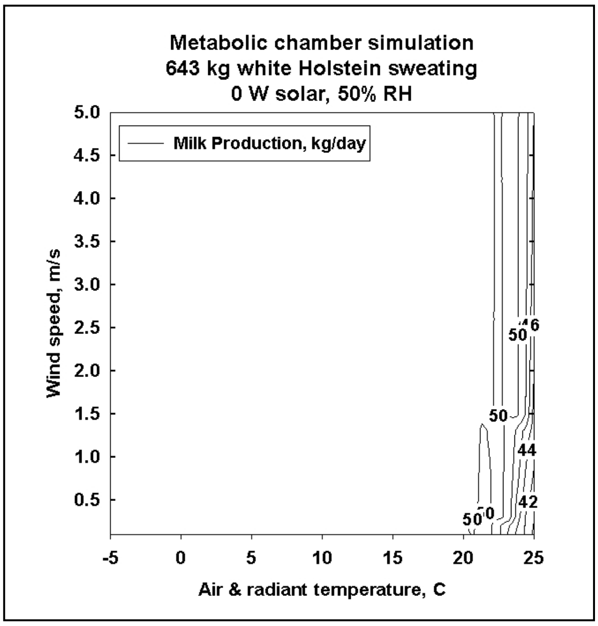

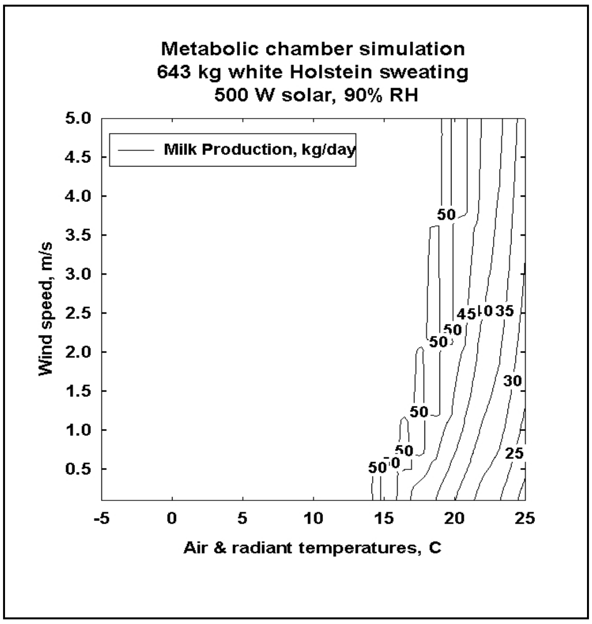

Figure S7c Figure S7d

"THI effects" of solar radiation and wind speed. The addition of solar radiation at 3 m/s moves the edge of the "cliff" back to the left to lower tolerated air temperatures.

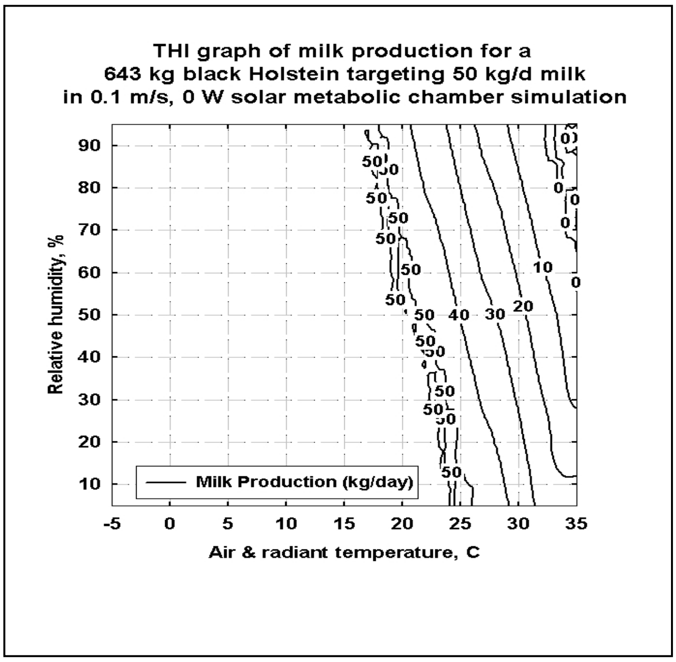

Figure S8a

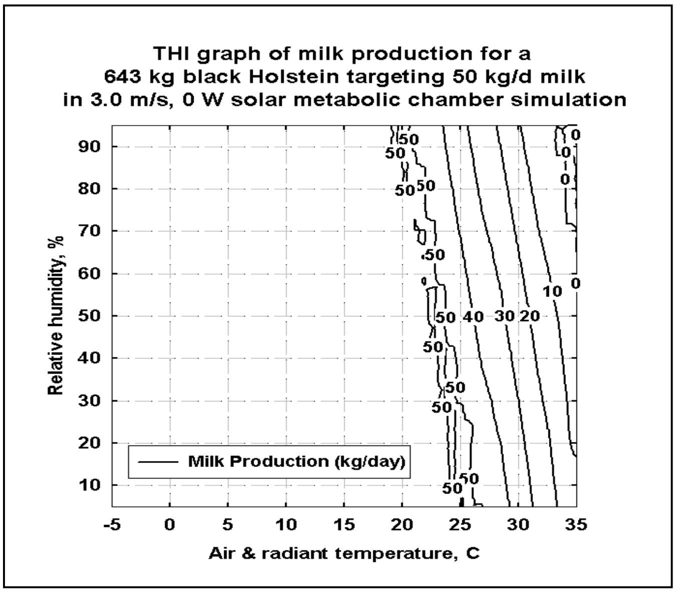

Figure S8b

Figure S8c looks the same at 2.9 m/s, but at 2.8 m/s, Figure S8d, there is a catastrophic drop in milk production potential that allows for less than 10% relative humidity for any milk production at all.
The reason for the sudden 'drop' turns out to be the level of solar radiation, 500 W/m^2, in the simulation.

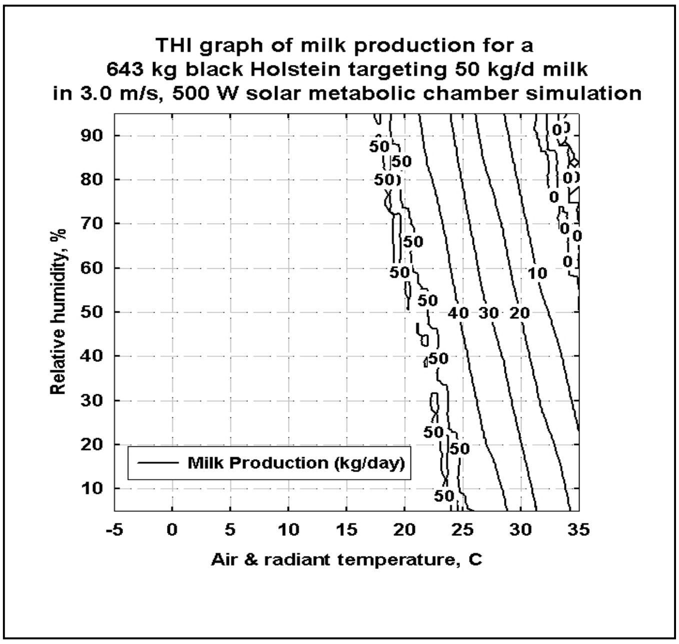

Figure S8c

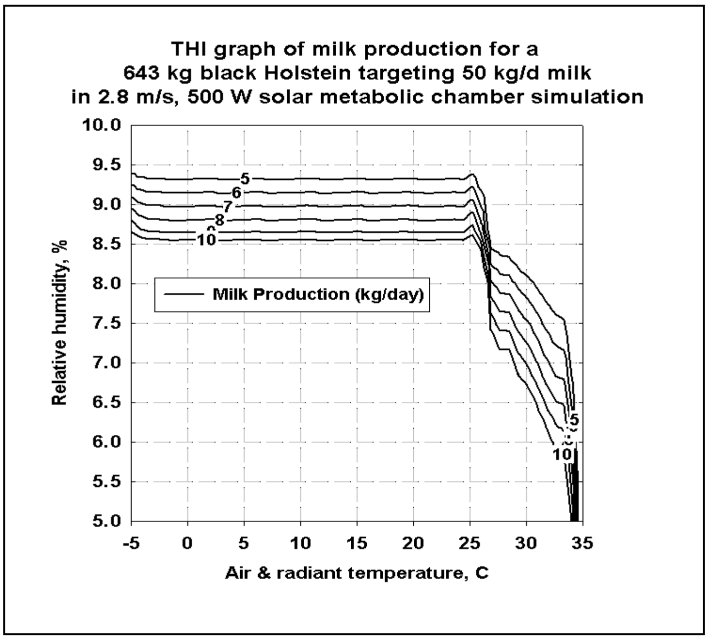

Figure S8d

Figure S8e shows that the 'plain' of milk production is achievable if the solar radiation is decreased to 480 W/m^2 at 2.8 m/s wind. Thus a 5th dimension of solar radiation, not typically visible in THI plots, is responsible for this apparent sudden loss of production capacity.

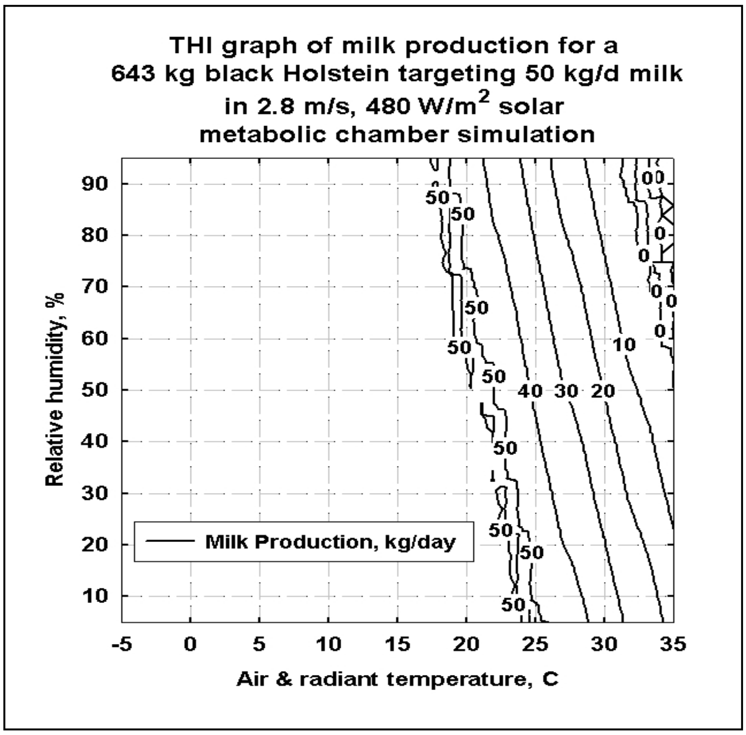

Figure S8e

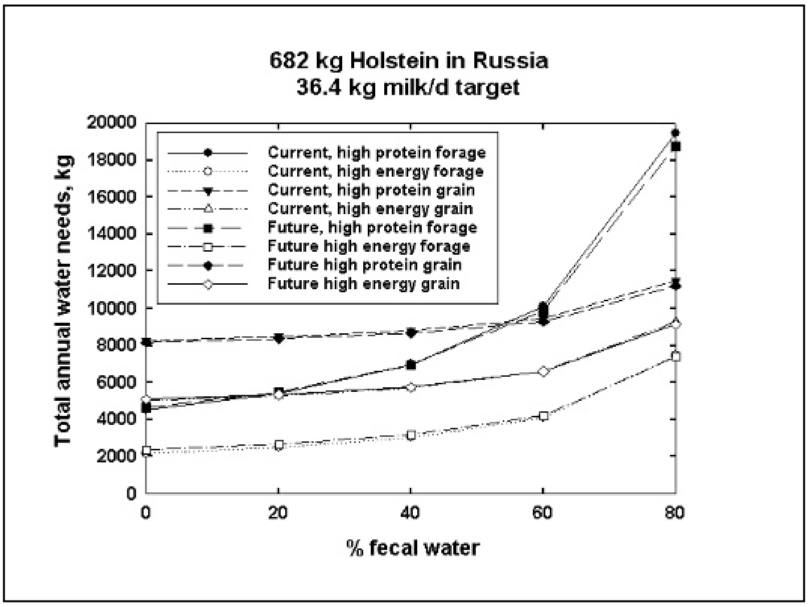

Figure S9a

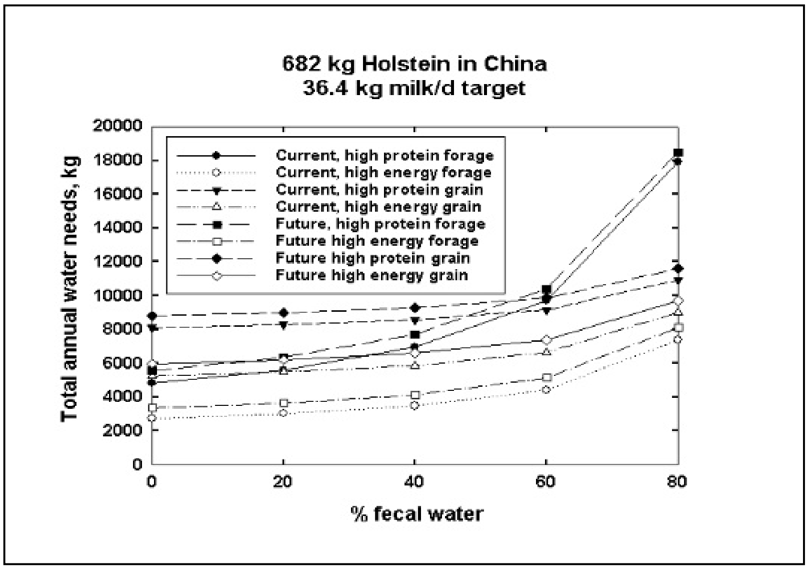

Figure S9b

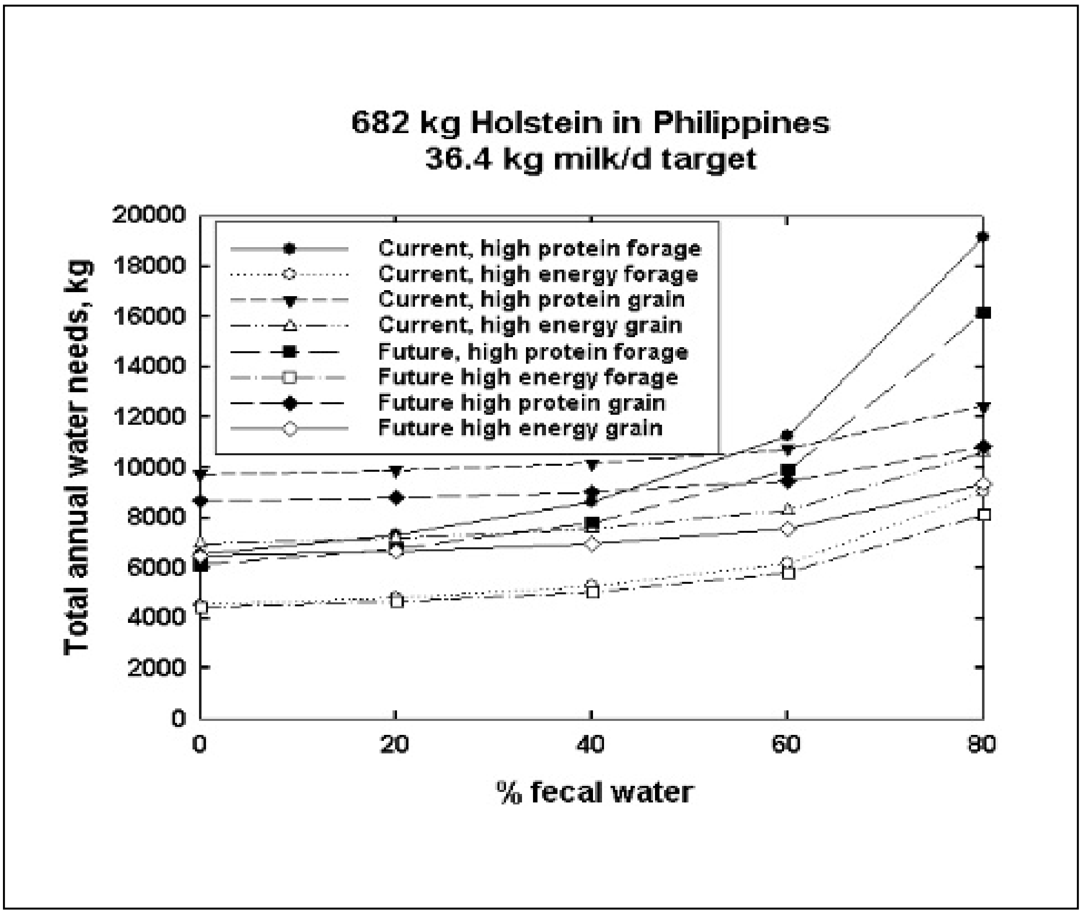

Figure S9c

Figures S9 a-c show the total annual water needs for a 50% black and 50% white free ranging Holstein weighing 682 kg at 60° north latitude (Russia), 30° north latitude (China), and 12° north latitude (Philippines) respectively from top to bottom. The annual water needs are impacted by percent fecal water, diet type and current versus future 3° warmer climates.
